# Targeted introduction of heritable point mutations into the plant mitochondrial genome

**DOI:** 10.1038/s41477-022-01108-y

**Published:** 2022-03-17

**Authors:** Joachim Forner, Dennis Kleinschmidt, Etienne H. Meyer, Axel Fischer, Robert Morbitzer, Thomas Lahaye, Mark A. Schöttler, Ralph Bock

**Affiliations:** 1 Max-Planck-Institut für Molekulare Pflanzenphysiologie, Potsdam-Golm, Germany; 2 ZMBP, Allgemeine Genetik, Universität Tübingen, Tübingen, Germany; 3 Present Address: Institut für Pflanzenphysiologie, Martin-Luther-Universität Halle-Wittenberg, Halle (Saale), Germany

**Keywords:** Genetic engineering, Plant molecular biology, Transgenic plants, Plant physiology, Molecular engineering in plants

## Abstract

The development of technologies for the genetic manipulation of mitochondrial genomes remains a major challenge. Here we report a method for the targeted introduction of mutations into plant mitochondrial DNA (mtDNA) that we refer to as transcription activator-like effector nuclease (TALEN) gene-drive mutagenesis (GDM), or TALEN-GDM. The method combines TALEN-induced site-specific cleavage of the mtDNA with selection for mutations that confer resistance to the TALEN cut. Applying TALEN-GDM to the tobacco mitochondrial *nad9* gene, we isolated a large set of mutants carrying single amino acid substitutions in the Nad9 protein. The mutants could be purified to homochondriomy and stably inherited their edited mtDNA in the expected maternal fashion. TALEN-GDM induces both transitions and transversions, and can access most nucleotide positions within the TALEN binding site. Our work provides an efficient method for targeted mitochondrial genome editing that produces genetically stable, homochondriomic and fertile plants with specific point mutations in their mtDNA.

## Abstract (web summary)

A method for targeted mutagenesis of mitochondrial genomes is presented. It combines site-specific DNA cleavage with selection for mutations that confer cleavage resistance, and produces genetically stable plants with edited mitochondrial genomes.

## Main

Basic and applied research on plant mitochondria continues to suffer from the lack of tools to engineer plant mitochondrial genomes. Similar to transgenic research with chloroplasts^1,2^, the availability of technologies for mitochondrial genome engineering would greatly facilitate functional genomics research (by reverse genetics—for example, ref. ^3^), allow the in vivo analysis of all steps in mitochondrial gene expression (for example, refs. ^4–6^) and enable the exploitation of mitochondria in biotechnology and synthetic biology^7,8^. Unfortunately, despite enormous efforts in the academic and industrial sectors, a technology for plant mitochondrial transformation is still not within reach. In addition to the lack of a mitochondria-specific selectable marker gene^9,10^, there are unknowns involved in the design of suitable expression cassettes (especially with regard to the largely enigmatic *cis*-elements for translation and translational regulation^11,12^), concerns about the low copy number of the mitochondrial genome^13^ and uncertainties about the efficiency of homologous recombination in mitochondria (although it is known that a homologous recombination activity exists^14–16^). In the absence of technologies for direct genetic transformation of the mitochondrial genome, indirect methods for introducing changes into mitochondrial DNA (mtDNA) of seed plants deserve serious consideration.

Genome editing tools have proven to provide powerful and nearly universally applicable methods for targeted mutagenesis^17–19^. Initially, these systems relied on site-directed DNA cleavage and were therefore restricted to the generation of insertions and/or deletions in the target sequence. Later, the combination of the DNA recognition properties of CRISPR–Cas systems with nucleoside deaminases produced new genome editing reagents, so-called base editors, that introduce specific point mutations without cutting the target DNA^20–23^. Since all CRISPR–Cas systems rely on RNA-mediated recognition of the target site in the genome by complementary base pairing, they cannot easily be adapted for mitochondrial genome editing. Although the import of RNA molecules, especially tRNAs, into mitochondria occurs in a number of organisms^24,25^, the import systems seem to have rather strict requirements for RNA structure, sequence and/or size^25,26^, and their utilization for the import of CRISPR RNAs has not yet been accomplished. In principle, CRISPR–Cas-based editing tools could be used in those few unicellular organisms (yeast, *Chlamydomonas reinhardtii*) where mitochondrial transformation is available, but they would not be very useful there, given that mitochondrial transformation occurs by homologous recombination^27,28^, which represents an even more powerful, versatile and precise genome editing and engineering tool than CRISPR–Cas. Instead, the great attraction of mitochondrial genome editing is that it could make non-transformable species amenable to site-specific sequence alterations in mitochondrial genes.

Given that CRISPR guide RNAs cannot readily be targeted to mitochondria, protein-only genome editing methods offer greater promise for mitochondrial genome engineering. Transcription activator-like effector nucleases (TALENs) targeted to mitochondria have been shown to be capable of inducing deletions in the mitochondrial genome^29^. Very recently, a base editing method originally developed for animal mitochondria^30^ has been tested in plant tissue culture and shown to be capable of introducing C-to-T mutations in chloroplasts and mitochondria^31,32^. However, genetically stable plants with a homogeneous population of mitochondrial genomes carrying the point mutation (referred to as homochondriomic plants) have not been obtained. Also, fertile base-edited plants that would transmit the genetic alteration in their mtDNA to the progeny have not been reported.

Here we describe the development of a method for the targeted introduction of stable point mutations into plant mitochondrial genomes. Combining site-specific genome cleavage by TALENs with a gene-drive-like selection strategy for mutations conferring resistance to TALEN cleavage, we have generated a large set of fertile homochondriomic mutant lines that harbour specific base substitutions in the mitochondrial NADH dehydrogenase subunit 9 (*nad9*) gene of tobacco, *Nicotiana tabacum*.

## Results

### TALEN-induced targeted cleavage of the mitochondrial genome

Transcription activator-like effectors (TALEs) recognize their target sequence by tandem arrays of 34 amino acid repeats that collectively form a superhelical structure. Each repeat pairs with a single base in the target sequence^33^. Base specificity is conferred by amino acid residues 12 and 13 of the repeat, which have been designated as the repeat variable diresidue. When TALE repeat arrays are fused to the catalytic domain of the restriction endonuclease FokI that induces DNA cleavage upon dimerization, TALENs designed to bind in tail-to-tail orientation on opposite strands of a given target sequence can induce DNA double-strand breaks at specific sites^34,35^.

To test whether TALENs targeted to mitochondria are capable of cleaving the tobacco mitochondrial genome, we selected the mitochondrial *nad9* gene as a target for genome editing. We chose *nad9* because it encodes a subunit of mitochondrial complex I, the NADH–ubiquinone oxidoreductase complex in the inner mitochondrial membrane, which is not essential for photoautotrophic growth^36,37^. This feature should allow the unbiased recovery of all mutations induced in the gene.

**Fig. 1:**
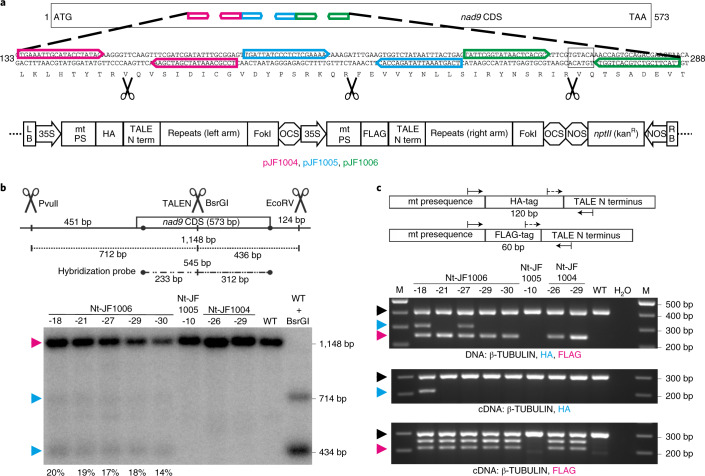
Generation of transgenic tobacco lines expressing TALENs targeted to the mitochondrial *nad9* locus. **a**, Binding sites of the three designed TALEN pairs in the *nad9* coding sequence (CDS) and schematic view of the binary vectors used for plant transformation. The nucleotide positions refer to the start codon (ATG = 1). The scissors indicate the predicted TALEN cut sites; the BsrGI restriction site is boxed. LB/RB, T-DNA left/right borders; 35S, CaMV 35S promoter; mt PS, mitochondrial presequence (from the *Arabidopsis* IVD protein); HA, 3xHA-tag; TALE N term, TALE amino terminus; Repeats, TALE DNA-binding units; FokI, endonuclease domain; OCS, octopine synthase terminator (*Agrobacterium*); FLAG, 3xFLAG-tag; NOS (octagon), nopaline synthase terminator (*Agrobacterium*); *nptII* (kan^R^), neomycin phosphotransferase II encoding kanamycin resistance; NOS (arrow), nopaline synthase promoter (*Agrobacterium*). The coloured arrows indicate TALEN binding sites. **b**, Detection of TALEN activity by Southern blot analysis. The predicted sizes of hybridizing restriction fragments and the location of the probe are depicted. The scissors mark the cut sites of the restriction enzymes PvuII, EcoRV and BsrGI, and of the pJF1006-encoded TALEN pair. BsrGI cuts 2 bp away from the predicted TALEN cut site. In addition to PvuII and EcoRV, BsrGI was included in a digestion reaction with wild-type DNA (WT + BsrGI; right) to simulate the TALEN cut. The numbers below the gel give the relative intensities of the TALEN cleavage products. The arrowheads mark the positions of the three expected signals (magenta indicates uncut; cyan indicates TALEN-cut). **c**, PCR and RT–PCR assays to test for the presence of TALEN arms in the lines shown in **b**. The binding sites of the PCR primers are shown in the upper panel (the dashed lines are for RT–PCR primers), and the lower panels display the PCR results. A sequence from the β-TUBULIN gene was amplified as an internal control. The arrowheads indicate the expected sizes for β-TUBULIN (black, 412 bp/303 bp for DNA/cDNA), the HA arm (cyan, 311 bp/210 bp) and the FLAG arm (magenta, 251 bp/212 bp). H_2_O, water control; M, DNA size marker. Only the FLAG–TALEN arm is present in Nt-JF1006-30. Pools of kanamycin-resistant T_1_ seedlings obtained by selfing were used for the nucleic acid extractions. See also Extended Data Fig. 4. The experiments in **b** and **c** were performed once.

**Extended Data Fig. 1:**
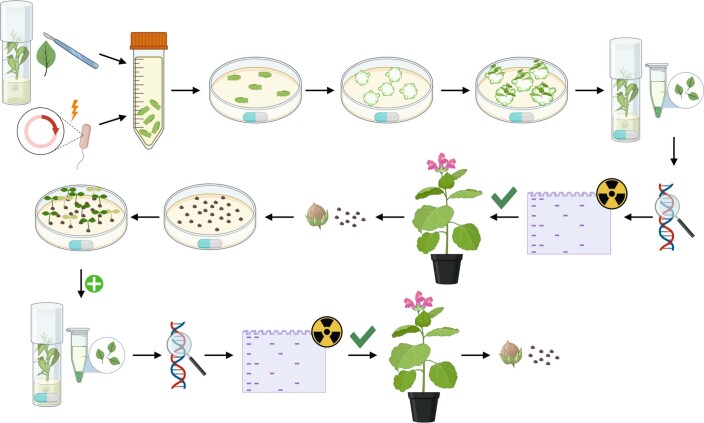
Isolation of TALEN-expressing lines. The workflow illustrating the procedures involved in the generation of transgenic TALEN-expressing lines and the detection of TALEN cleavage activity in the mitochondrial genome is schematically shown. Tobacco leaves are transformed with *Agrobacterium tumefaciens* cells harboring TALEN-encoding plasmids. Putative transgenic T_0_ shoots are regenerated on medium containing kanamycin, rooted and transferred to the greenhouse. DNA is isolated from these plants and assayed by Southern blotting for TALEN activity (that is, cleavage of *nad9*). Individual T_1_ seedlings derived from these plants (and selected for kanamycin resistance) are screened again for TALEN activity and then used for T_2_ seed production. The blue-white pill indicates presence of kanamycin in the medium.

**Extended Data Fig. 2:**
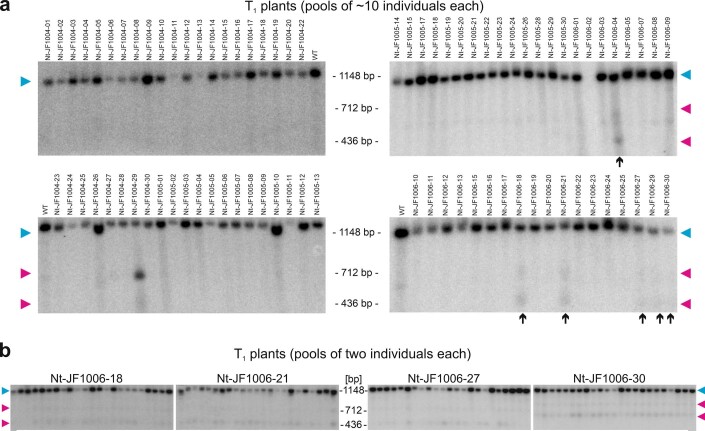
Southern blot analyses to identify lines with stable functional TALEN expression. For details, see Fig. 1b. (**a**) The blots in the upper panel show the full screen of all transgenic plants obtained with the three TALEN constructs. Pools of approximately ten kanamycin-resistant T_1_ seedlings were used for DNA extraction. Candidate lines showing a hybridization pattern consistent with TALEN cleavage activity are marked by black arrows. (**b**) Analysis of additional T_1_ descendants of four candidate T_0_ lines. Each lane represents a pool of two individual plants. The 1148 bp fragment (*nad9* not cut by TALENs) is marked by a cyan arrowhead, the two fragments resulting from the TALEN cut (712 bp and 436 bp) are indicated by magenta arrowheads. All Southern blots shown here were performed once.

Three anti-*nad9* mitochondrial TALEN pairs were designed, and the corresponding gene sequences were inserted into vectors for the transformation of the plant nuclear genome (Methods and Fig. 1a). The target sequences of the TALENs are 19 base pairs (bp) long and should be more than sufficient to confer target-site-specific cleavage in the comparably small mitochondrial genome without causing off-target effects. A mitochondrial presequence encoding a transit peptide for protein import into the mitochondrial compartment was added, and distinct epitope tags (HA and FLAG) were inserted to facilitate the detection of the two TALEN proteins (HA for the ‘left’ and FLAG for the ‘right’ TALEN) in transgenic plants. The three constructs (pJF1004, pJF1005 and pJF1006; Fig. 1a) were introduced into tobacco (*Nicotiana tabacum*) cells by *Agrobacterium*-mediated transformation, and approximately 30 transgenic lines per construct were selected (Extended Data Figs. 1 and 2). All T_0_ plants were phenotypically inconspicuous and were indistinguishable from wild-type plants when grown under standard greenhouse conditions.

Initial genotyping of the lines by DNA sequencing of the *nad9* locus revealed no indication of the presence of mutations (insertions, deletions or single nucleotide polymorphisms (SNPs)) at or near the predicted TALEN cut sites. To test whether cleavage of the mitochondrial genome occurred, Southern blot analyses with *nad9*-specific probes were conducted. Several lines (all from the same construct, pJF1006) displayed additional hybridization signals whose sizes were consistent with the predicted sizes of TALEN cleavage products (Fig. 1b and Extended Data Fig. 2a). The same hybridizing fragments were consistently observed in all T_1_ progeny of these lines (Extended Data Fig. 2b).

**Extended Data Fig. 3:**
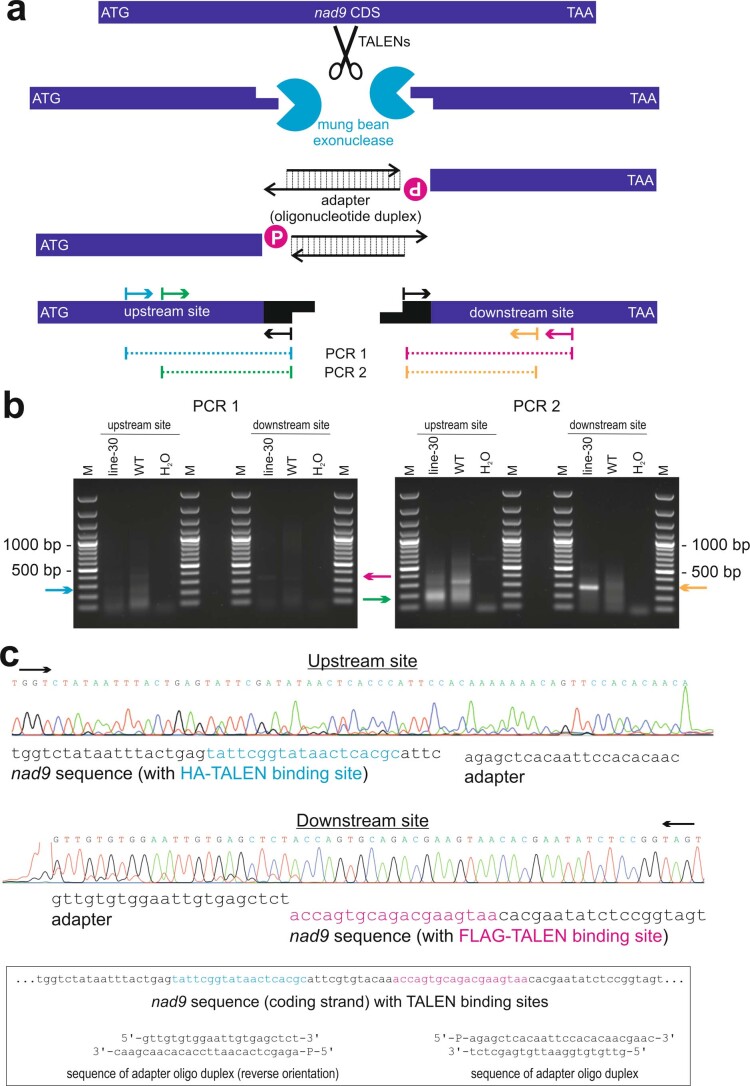
Detection of TALEN-induced DNA double-strand breaks. (**a**) End-mapping strategy to verify that the TALENs cut at the predicted sites. Arrows indicate oligonucleotides or primer-binding sites. mtDNA cut by the TALENs *in vivo* is blunted by mung bean exonuclease *in vitro*, followed by adapter ligation and PCR amplification. Note that PCR 2 is a half-nested PCR using the products of PCR 1 as template. P, 5’ monophosphate group of oligonucleotides. (**b**) PCR results obtained from execution of the strategy depicted in panel (a). Arrows indicate the expected sizes of products derived from TALEN-cut mtDNA. Cyan arrow, expected size (270 bp) of the amplification product of upstream site PCR 1; magenta arrow, expected product size (350 bp) for downstream site PCR 1; green arrow, expected amplicon size (192 bp) for upstream site PCR 2; orange arrow, expected amplicon size (288 bp) for downstream site PCR 2; line-30, line Nt-JF1006-30; WT, wild-type control; H_2_O, water control; M, DNA size marker. One of two similar experiments with similar results is shown. (**c**) Sequencing chromatograms for the products of PCR 2. The chromatograms show the DNA sequence obtained from sequencing the PCR products marked by the green (upstream site) and orange (downstream site) arrows, respectively, in panel (b). For comparison, the sequences of the adapter oligonucleotide duplex and the *nad9* TALEN target site are also given (boxed). Black arrows indicate the direction of the sequencing reactions.

To ultimately confirm that the additional hybridizing fragments originate from TALEN cleavage of the mitochondrial genome, we attempted to map the ends of the fragments. For this purpose, extracted total plant DNA was treated with mung bean exonuclease to remove single-stranded overhangs from double-strand breaks, followed by ligation to double-stranded DNA adapters (Extended Data Fig. 3a; see the Methods for the details). Subsequent PCR amplification of the ligation products and DNA sequencing indeed revealed cleavage of the *nad9* locus at the TALEN binding sites (Extended Data Fig. 3b,c).

**Extended Data Fig. 4:**
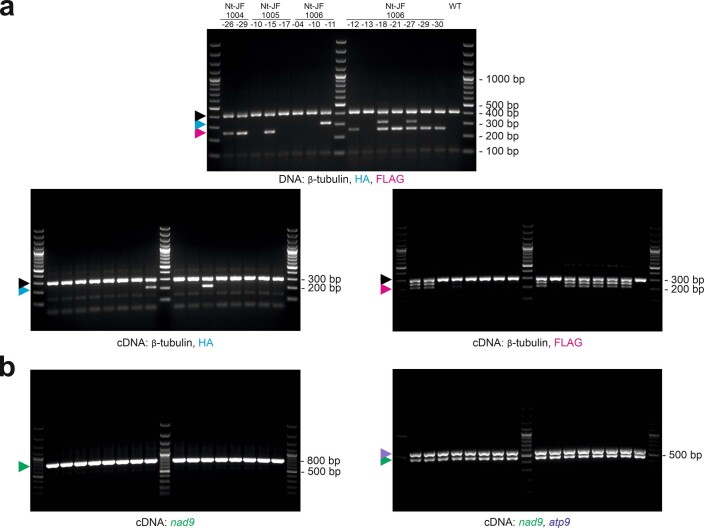
Tests for presence of HA- and FLAG-TALEN arms, and analysis of *nad9* expression in a subset of Nt-JF1004, Nt-JF1005 and Nt-JF1006 lines. (**a**) Extension of the analyses shown in Fig. 1c to a larger set of plants. See legend to Fig. 1c for details. (**b**) Analyses of the same plants as shown in (a) by amplification of full-length *nad9* cDNA with primers oJF311 and oJF748 (yielding a product of 769 bp, indicated by the green arrowhead; left panel) for subsequent analysis by Sanger sequencing, and partial *nad9* cDNA with primers oJF496 and oJF748 (465 bp, indicated by the green arrowhead; right panel) and full-length *atp9* cDNA with primers oJF1145 and oJF748 (568 bp, purple arrowhead) for semiquantitative analysis of expression strength. Primers for first strand cDNA synthesis were oJF1113 (*nad9*) and oJF1144 (*atp9*). Sequencing analyses showed all full-length *nad9* RT-PCR products to be fully edited and free of mutations. The loading scheme is identical for all five gels. For primer sequences, see Extended Data Table 2. The experiments shown in (a) and (b) were performed once.

The hybridization intensities of the two TALEN cleavage products relative to the signal intensity of the uncut genome were remarkably similar in all transgenic lines that displayed detectable TALEN activity (Fig. 1b and Extended Data Fig. 2), possibly suggesting that the observed level of cleavage is close to the maximum tolerable by the plant and/or represents the achievable balance of double-strand break and repair. Interestingly, genotyping of the lines revealed that three out of five Nt-JF1006 lines that showed TALEN-induced cleavage of the mitochondrial genome did not harbour both TALEN genes. Instead, they contained the gene for only one of the two TALEN arms (Fig. 1c and Extended Data Fig. 4), although this did not appreciably affect TALEN cleavage efficiency. This observation is in line with previous research demonstrating that (1) homodimeric TALENs function efficiently in yeast^38^, and (2) a DNA-bound FokI monomer can recruit a second monomer from solution to facilitate the cleavage of double-stranded DNA^39^. Since one TALEN arm was obviously sufficient to induce genome cleavage in the mtDNA, most subsequent experiments were conducted with a line (Nt-JF1006-30; Fig. 1b,c) that harbours only one TALEN gene (encoding the ‘right’ arm; Fig. 1). All mutants presented here are derived from that line.

TALEN cleavage of nuclear DNA typically results in short insertions and/or deletions, due to imperfect double-strand break repair. Our failure to detect such mutations at the *nad9* TALEN cleavage site is consistent with the previously proposed absence of a religation pathway of DNA double-strand break repair from both plastids^40^ and plant mitochondria^29^. Instead, double-strand breaks in plant organellar genomes seem to be repaired by homologous recombination or, in rare instances, by microhomology-mediated illegitimate recombination^29,40^.

### A gene-drive-like strategy for the induction of point mutations

Given the likely absence of (error-prone) religation of TALEN-induced cuts in the mtDNA and the remarkably stable low-level genome cleavage frequency in all transgenic lines expressing active TALENs (Fig. 1b and Extended Data Fig. 2), we reasoned that any point mutation in the TALEN binding site within *nad9* should confer a strong selective advantage in that it would make the mutated mitochondrial genome less susceptible to the TALEN cut. This is because repair events that restore the wild-type sequence remain TALEN targets and will be cleaved again. By contrast, repair products that contain mutations at the TALEN target site should become fixed since they are no longer TALEN substrates. Continued cleavage activity of TALENs on the wild-type sequence is thus expected to select for mutant alleles that weaken TALEN binding to the target sequence.

**Fig. 2:**
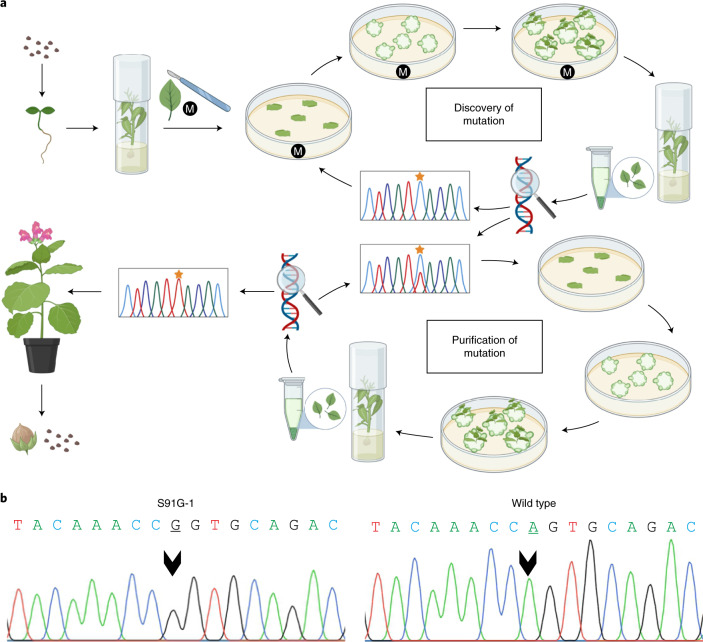
Workflow for the isolation of tobacco lines with TALEN-induced point mutations in the mitochondrial *nad9* gene. **a**, Schematic overview of the experimental procedures. Transgenic plants with confirmed TALEN activity (Extended Data Fig. 1) were raised from seeds, and leaves were harvested, cut into pieces and placed onto shoot induction medium. Leaves from regenerated shoots were genotyped for mutations in *nad9* (‘Discovery of mutation’). If no mutation was detected, a new regeneration round was initiated. When a point mutation was found, an additional regeneration round was conducted to promote genome segregation and facilitate the isolation of homochondriomic lines (‘Purification of mutation’). Homochondriomic mutant shoots were rooted and transferred to the greenhouse for seed production. In some experiments, the mutagens (M) EtBr or NEU were added to the shoot induction medium or applied during seed imbibition. See the text, the Methods and the Supplementary Methods for the details. **b**, Exemplary sequencing chromatograms showing the successful isolation of a homochondriomic *nad9* mutant carrying a Ser-to-Gly exchange in amino acid position 91 of the Nad9 protein (corresponding to an A-to-G substitution in nucleotide position 271 of the *nad9* reading frame). The mutated position is indicated by arrowheads in the sequencing chromatograms of the S91G-1 mutant line and the wild type. The sequencing primer was oJF271 (Supplementary Table 2).

To set up such a gene-drive-like system, we performed repeated regeneration rounds in tissue culture to increase the chances of mutant recovery by single-cell passage of mtDNA (Fig. 2a). To this end, three T_1_ seedlings each from five TALEN-active lines (Fig. 1b) were passed through ten consecutive cycles of regeneration and selection. In each round, regenerating shoots were assayed by sequencing their *nad9* locus and were subsequently used for the next regeneration round. After the sixth round, a single mutant was recovered from the TALEN-expressing line Nt-JF1006-30 (Fig. 1b). The line harboured an A-to-G point mutation at position 271 of the *nad9* coding region (A271G; Fig. 2b), leading to an amino acid substitution from serine to glycine (S91G). The corresponding mitochondrial mutant line was named S91G-1 (as the first line recovered with this particular point mutation).

Assuming that TALEN activity was not causally responsible for the appearance of the point mutation but rather selected for it through the above-described gene-drive-like mechanism, we reasoned that the application of mutagens should greatly promote the recovery of TALEN-resistant mitochondrial mutants (Fig. 2). We therefore exposed seeds and leaf explants to two mutagens that have been shown to specifically affect organellar genomes: *N*-nitroso-*N*-ethylurea (NEU^41^) and ethidium bromide (EtBr^42^). Seed imbibition in 5 mM NEU or 0.2% EtBr did not result in increased recovery of *nad9* mutants. In each experiment, 50 T_1_ seedlings were sequenced, but no mutations in *nad9* were found. Next, we conducted three successive rounds of plant regeneration with leaf explants that either had been dipped into NEU solution prior to their exposure to regeneration medium or were regenerated in the presence of EtBr (Fig. 2a). NEU dipping did not result in a substantial increase in *nad9* mutant recovery. Only a single mutant was identified from 76 sequenced regenerated shoots (Table 1 and Supplementary Table 1). By contrast, plant regeneration in the presence of EtBr strongly increased the appearance of *nad9* mutations within the TALEN target sequence, and six new mutants could be identified by the sequencing of 94 regenerants (Table 1 and Supplementary Table 1). Comparable numbers of mutants were identified from cultures kept in constant light and cultures exposed to a light–dark cycle (Table 1), suggesting that the light regime does not strongly affect the selection efficiency of mitochondrial mutants.

**Table 1:** Effects of light conditions and mutagen application on the recovery of mitochondrial mutants

	16 h light / 8 h dark			24 h light / 0 h dark		
Treatment	None	EtBr	NEU	None	EtBr	NEU
1st reg. round	0/10	2^a^/10	0/10	0/10	3^b^/10	0/10
2nd reg. round	0/10	0/10	0/10	0/10	0/10	0/10
3rd reg. round	0/22	0/16	1^c^/19	0/24	1^d^/38	0/20
Total	0/42	2/36	1/36	0/44	4/58	0/40

The numbers give the number of mutants obtained per number of regenerated shoots assayed. The concentration of EtBr was 0.001%, and that of NEU was 5 mM; reg. round, regeneration round.

^a^Lines S91G-2 and D93E/E94K-1.

^b^Initially, two mutations were obtained. One gave rise to line Q89K-1; the other (T90N-1) was lost due to random mitochondrial genome segregation. However, one regenerated shoot later displayed a new mutation (line D93G-1) that was recovered.

^c^Line V95I-1.

^d^Line R85L-1.

### Homochondriomy of *nad9* mutants

**Fig. 3:**
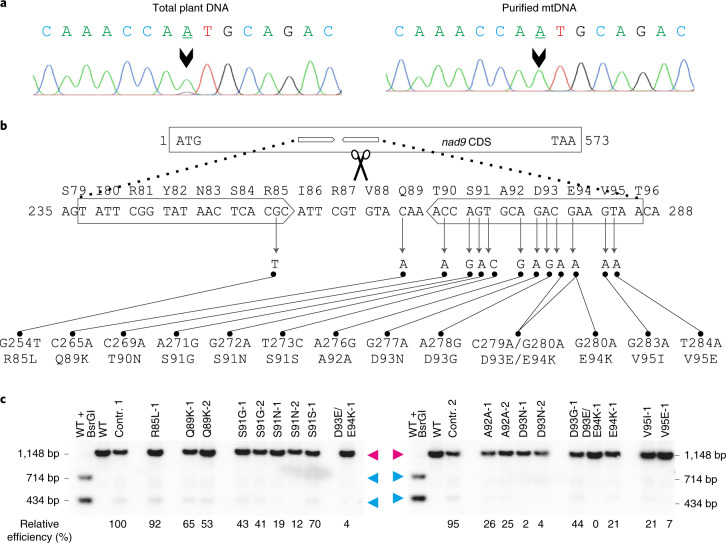
Apparent heterochondriomy, locations of the TALEN-induced point mutations in *nad9* and effects of the point mutations on TALEN cleavage activity. **a**, Genotyping of an S91N-1 mutant plant carrying the G272A mutation. Sequencing of PCR products amplified from total plant DNA (with primers oJF271 and oJF272; Supplementary Table 2) suggested persistent heteroplasmy (that is, the presence of residual copies of wild-type *nad9* alleles), as evidenced by an A + G double peak (arrowhead, left sequence). However, when the same PCR product was generated from purified mtDNA as a template, a clean single A peak is seen (right sequence). The sequencing was done with primer oJF271. **b**, Overview of all TALEN-induced mutations obtained in *nad9*. The nucleotide positions in the *nad9* coding sequence (ATG = 1) and the amino acid positions in the Nad9 protein are given. The block arrows denote the pJF1006 TALEN binding sites. The ‘left’ (HA-)TALEN (not present in line Nt-JF1006-30) extends from nucleotide positions 237 to 255 and the ‘right’ (FLAG-)TALEN from positions 268 to 286. The scissors point to the predicted TALEN cut site. **c**, Analysis of TALEN cleavage activity by Southern blotting using pools of kanamycin-resistant seedlings (from back-crosses of the original mutants with the wild type). The same restriction enzymes and hybridization probe as in Fig. 1b were used, but electrophoretic separation was done in a 2% agarose gel. To determine the relative cutting efficiencies, the percentage of cut *nad9* was calculated for each lane and then divided by the respective value for control line 1 (contr. 1). The smaller of the two *nad9* cleavage products was used for quantification, because it is covered by a larger portion of the hybridization probe. Contr. 1 and contr. 2 are descendants of Nt-JF1006-30 plants having gone through the same mutagenesis procedure as the point mutants, but retaining a wild-type *nad9* sequence. Line D93E/E94K-1 is represented twice (descendants of two different vegetative clones of the original mutant plant). The full Southern blot with enhanced contrast settings is presented in Extended Data Fig. 5. Unlike all other mutants, line R85L-1 may not be the result of a gene drive effect. A technical replicate of the Southern blot yielded similar results.

The suspected gene-drive-like effect should also strongly accelerate the attainment of the homochondriomic state. Homochondriomy refers to the presence of a homogeneous population of mutated mitochondrial genomes and the absence of residual wild-type copies of the (polyploid) mtDNA^13^. Indeed, nearly all of our mitochondrial mutants quickly reached a state in which the mutated genome represented the prevailing genome type. Typically, upon initial detection of the mutation by DNA sequencing of the *nad9* locus, the mutated nucleotide already accounted for >50% of the peak intensity in the sequencing chromatogram (Fig. 3a). However, a weak signal for the wild-type nucleotide persisted even after multiple additional cycles of plant regeneration and even after multiple rounds of crosses and sexual propagation.

Previous work on plastid transformation has shown that weak wild-type-like signals (detected by DNA sequencing and/or Southern blot hybridization) are not due to persistent heteroplasmy but are rather explained by the presence of promiscuous plastid DNA sequences in the nuclear genome that originate from rampant gene transfer from the plastid to the nuclear genome^43–46^. This explanation (and the homoplasmy of the transplastomic plants) was ultimately confirmed by chloroplast isolation and analysis of purified plastid DNA^47,48^. As mtDNA sequences are known to also frequently escape to the nuclear genome^49–51^, we reasoned that the small peak for the wild-type nucleotide in our *nad9* mutants (Fig. 3a) may come not from persistent heterochondriomy but rather from promiscuous mitochondrial *nad9* sequences that reside in the tobacco nuclear genome. To confirm this hypothesis, we isolated mitochondria from mutant plants and sequenced the *nad9* locus from purified mtDNA. This analysis revealed the complete absence of the wild-type peak (Fig. 3a), indicating the presence of a homogeneous population of mutated mitochondrial genomes (homochondriomy).

The *nad9* mutant plants flowered readily and produced abundant seeds. To ultimately confirm homochondriomy, mutant plants were selfed and crossed to wild-type plants. The *nad9* mutations were uniformly present in the progeny of selfed mutants and all crosses using a mitochondrial mutant as the maternal parent, in line with the maternal inheritance of the mitochondrial genome^52^. Deep sequencing of *nad9* products from PCR with reverse transcription (RT–PCR) (which allowed the exclusion of promiscuous *nad9* sequences in the nucleus based on the absence of RNA editing) further confirmed the homochondriomic state of the mutations (Supplementary Data 1).

Taken together, these data suggest that, in our selection system (Fig. 2), mitochondrial mutations attain the homochondriomic state very quickly, presumably due to the strong selective advantage conferred by mutations in the TALEN binding sites (Fig. 3c) and the resulting gene-drive-like mechanism. We therefore refer to our TALEN-based mutagenesis method as TALEN-gene-drive mutagenesis (TALEN-GDM).

### Characterization of *nad9* mutants

**Extended Data Fig. 5:**
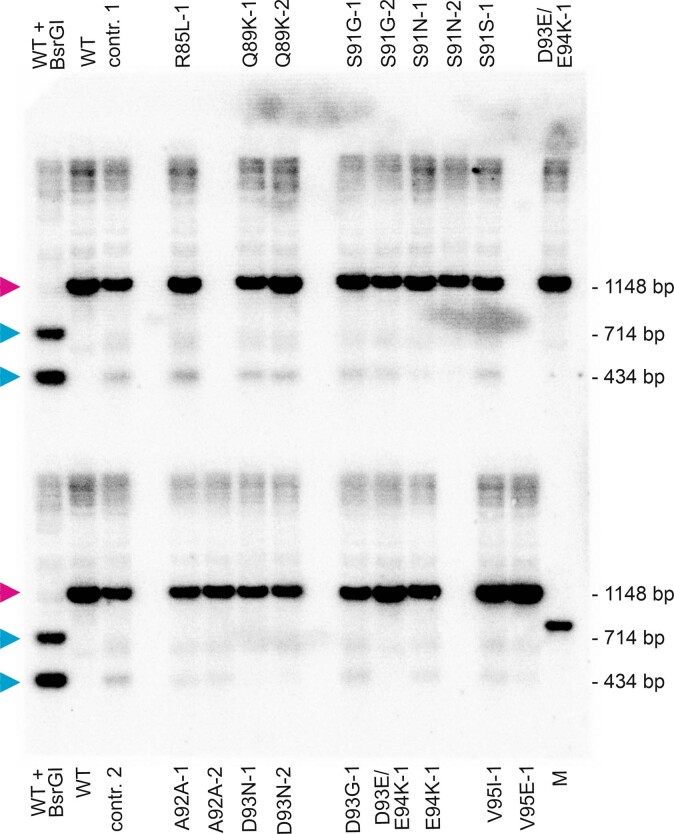
Southern blot from Fig. 3c shown with enhanced contrast to better visualize weak hybridization signals. M, DNA size marker (with the 800 bp marker fragment cross-hybridizing to the probe).

In the experiments described above (Table 1) and additional sets of experiments that are summarized in Supplementary Table 1, a total of 20 independent *nad9* mutants were isolated (Fig. 3b and Supplementary Table 1). Some point mutations were recovered independently up to three times (Supplementary Table 1). A wide spectrum of mutations (including both transitions and transversions) was obtained, indicating that the method has no pronounced bias in the mutation types that can be recovered. As expected, the mutations clustered in the TALEN binding site, although two point mutations were located a few nucleotides outside of the binding motif. It seems likely that at least one of these mutations (C265A = Q89K) also weakens TALEN binding to the target sequence, as evidenced by quantitation of the TALEN cleavage activity in the mutant *nad9* sequences (Fig. 3c and Extended Data Fig. 5), whereas the other one (G254T = R85L) may be the only mutation that was not obtained by a (pronounced) gene drive effect. This R85L mutant was also the only line that yielded many regenerating shoots (9 out of 22) that displayed only the wild-type *nad9* allele when the initial heterochondriomic mutant plant was subjected to a new regeneration round—something never observed for any of the other mutants.

A double mutant of two adjacent nucleotides (C279A and G280A; Fig. 3b) was also obtained. The two mutations affect neighbouring codons and are both non-synonymous substitutions, thus resulting in two adjacent amino acid exchanges in Nad9 (D93E and E94K; Fig. 3b). The two nucleotide substitutions may have arisen independently (in that each conferred a selective advantage by reducing the sensitivity of the genome to TALEN cleavage), or they were introduced together upon imperfect repair of a TALEN cut by homologous recombination.

**Extended Data Fig. 6:**
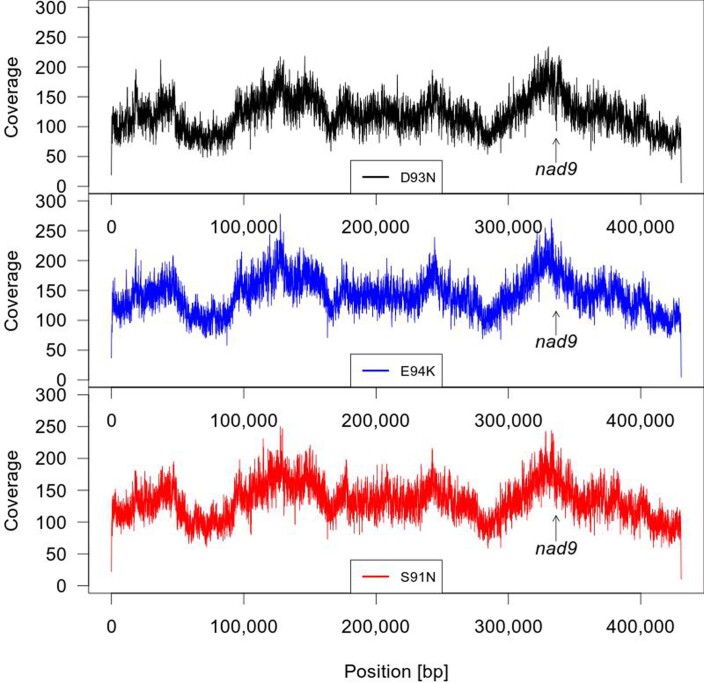
Read coverage across the whole mitochondrial genome in next-generation sequencing experiments. The mitochondrial genomes of mutant lines D93N-1, E94K-1 and S91N-1 were sequenced. Nucleotide positions [bp] on the x-axis are according to the tobacco mitochondrial reference genome sequence (NC_006581). The y-axis shows the number of reads per position. Upper panel: D93N-1 (black), middle panel: E94K-1 (blue), lower panel: S91N-1 (red). The arrow indicates the position of *nad9*.

All *nad9* mutants were crossed using the wild type as the pollen donor to eliminate the transgenes from the nuclear genome. The plants were also characterized at the complementary DNA level (by sequencing the entire *nad9* coding region and parts of the 5′ and 3′ untranslated regions) to assay for the presence of off-target mutations or possible defects in *nad9* mRNA editing that, in theory, could result from the introduced point mutations. No off-target mutations in *nad9* or editing defects were found in any of the mutant lines, suggesting high specificity of the TALEN-GDM method for the targeted sequence in the mitochondrial genome. To confirm the specificity of TALEN-GDM at the genome-wide level, three mutant lines were additionally subjected to next-generation sequencing (NGS) of the whole mitochondrial genome (Supplementary Data 2 and Extended Data Fig. 6). No mutations other than the point mutations in *nad9* were detected in any of the three sequenced genomes. Twelve SNPs compared to the reference genome (NC_006581.1) from cultivar Bright Yellow 4 were found in all three mutant lines, but all of these are also present in the wild type of our cultivar, Petit Havana, and thus represent intraspecific variation rather than off-target mutations. Furthermore, no sequences corresponding to the wild-type *nad9* allele were detected in the mutants (with nearly 200× coverage), further confirming homochondriomy.

**Extended Data Fig. 7:**
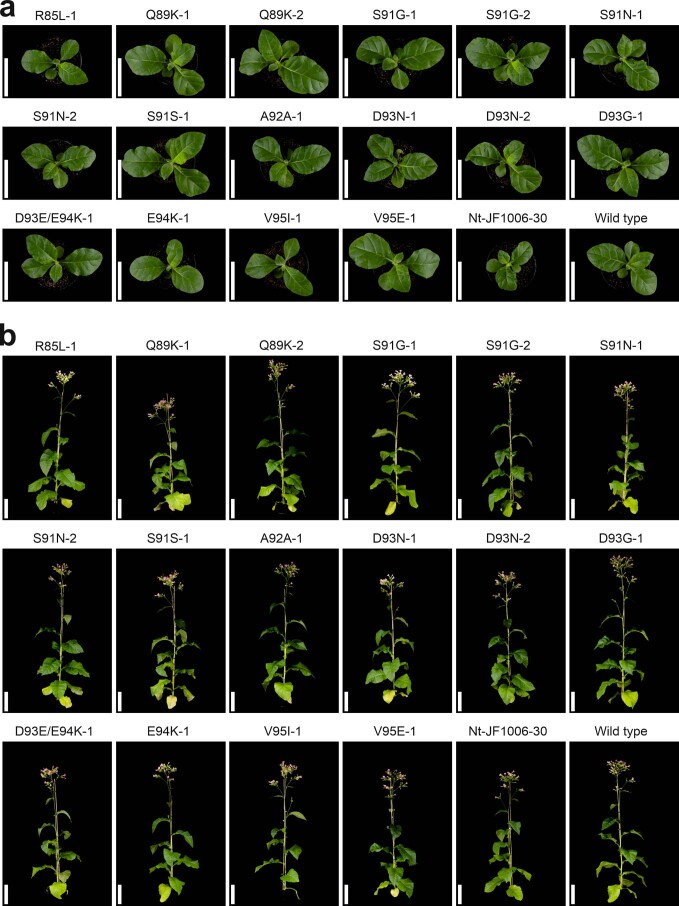
Phenotypes of the full set of *nad9* mutant plants. (**a**) Photographs taken 33 days after sowing. Plants were raised from seeds, pre-grown for 30 days in a nursery chamber, and then transferred to the greenhouse. Line names indicate the amino acid substitutions in Nad9. Scale bars: 10 cm. Nt-JF1006-30, TALEN control line with wild-type *nad9*. (**b**) The same plants as in (a) photographed 61 days after sowing. Scale bars: 20 cm. The images of E94K-1 and the wild type are as in Fig. 4c,d.

**Extended Data Fig. 8:**
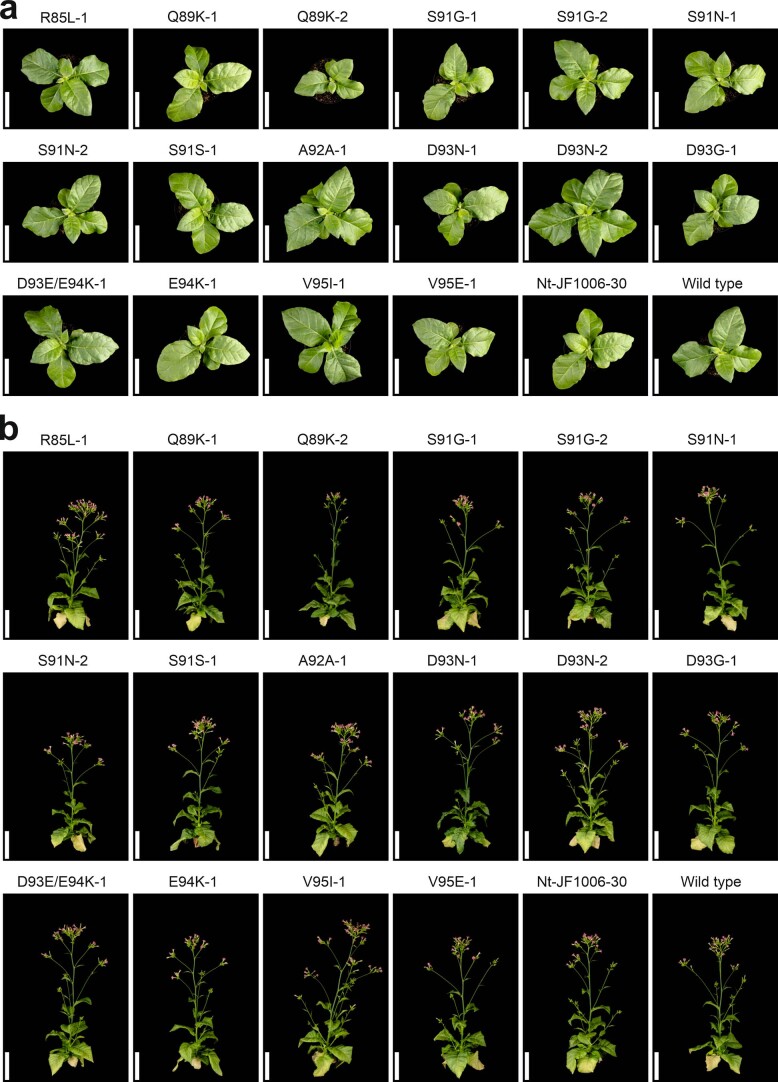
Phenotypes of the full set of *nad9* mutant lines under high-light conditions (cf. Extended Data Fig. 7). (**a**) Photographs taken 40 days after sowing. Plants were raised from seeds, pre-grown for 28 days in a nursery chamber, and then transferred to a growth chamber with a light intensity of 1000 µE m^−2^ s^−1^. Scale bars: 10 cm. Nt-JF1006-30, TALEN control line with wild-type *nad9*. (**b**) The same plants as in (a) photographed 63 days after sowing. Scale bars: 20 cm.

Next, we analysed the phenotypes of the *nad9* mutants. To this end, the entire set of mutants was grown under standard greenhouse conditions and also under high-light conditions (Methods) to promote maximum growth rates. No obvious phenotypes were detected, and all mutants were indistinguishable in growth and development from the wild-type controls (Extended Data Figs. 7 and 8). These observations are in line with the surface-exposed location of the targeted Nad9 domain in complex I and its unlikely involvement in complex biogenesis^37^.

**Fig. 4:**
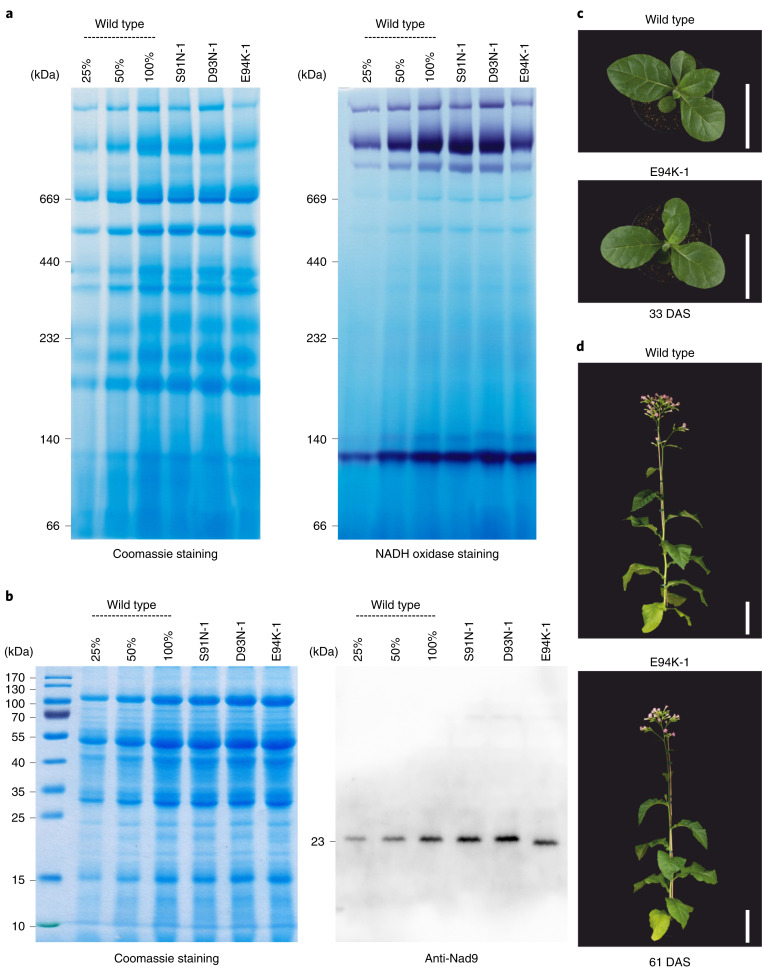
Biochemical characterization and phenotypes of mitochondrial *nad9* mutants. **a**, Analysis of mitochondrial protein complexes and NADH oxidase (complex I) staining in selected *nad9* mutants by blue-native polyacrylamide gel electrophoresis (BN–PAGE). Protein sizes are given in kDa. A dilution series of the wild-type sample (25%, 50% and 100%) was loaded to allow for semiquantitative assessment of protein accumulation. A technical replicate of the BN–PAGE (stained with Coomassie) yielded similar results; the NADH oxidase (complex I) staining was performed once. **b**, Accumulation and electrophoretic mobility of Nad9 proteins in *nad9* mutants assessed by SDS–PAGE. Note the faster migration of the E94K variant of Nad9. The Coomassie-stained gel is shown to confirm equal loading. The SDS–PAGE (including Coomassie staining) was done seven times and the anti-Nad9 western blot five times (technical replicates), with similar results (see also Extended Data Fig. 9). **c**, Plant phenotypes. Plants were grown on soil and cultivated in long-day conditions in the greenhouse. A wild-type plant and an E94K-1 mutant plant (harbouring a G280A mutation in *nad9*) are shown. The photographs were taken 33 days after sowing (DAS). Scale bars, 10 cm. **d**, Plants photographed 61 DAS. Scale bars, 20 cm.

**Fig. 5:**
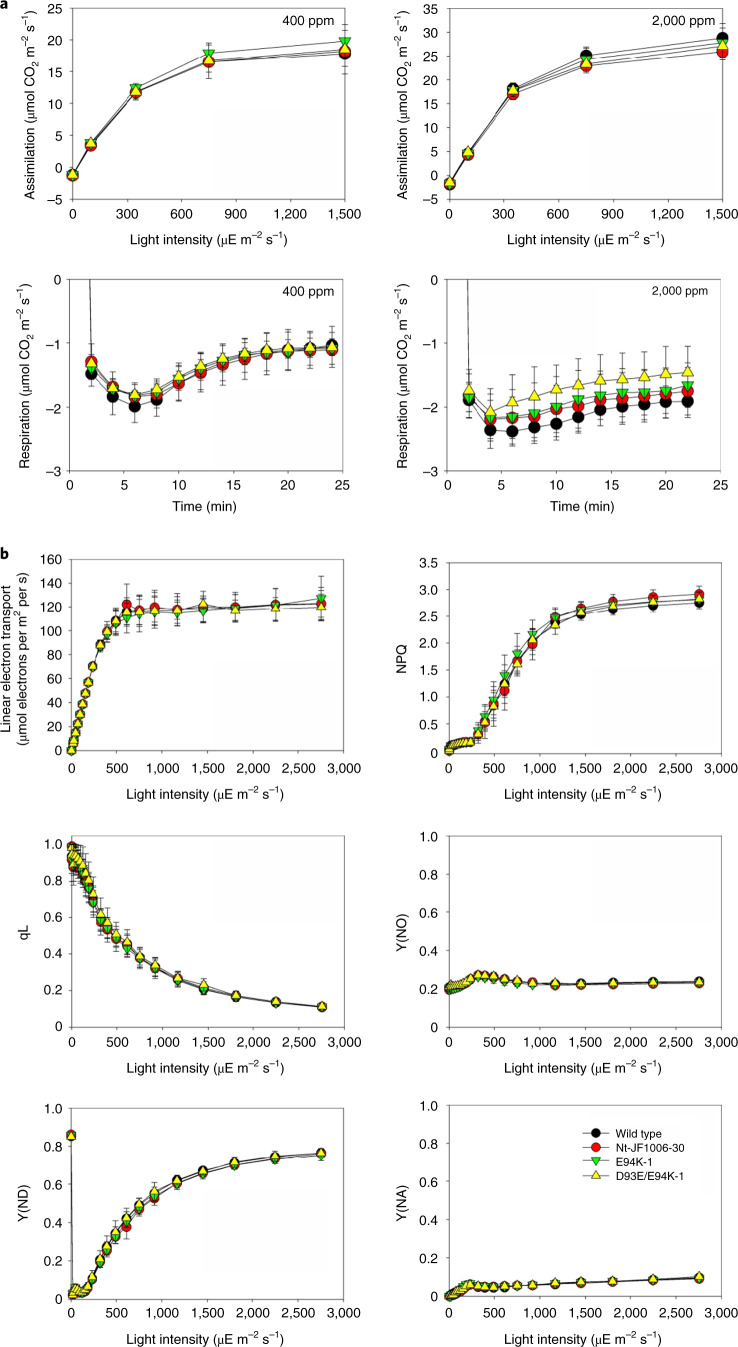
Determination of gas exchange rates and photosynthetic parameters of two mitochondrial mutants grown at 350 µE m^−2^ s^−1^ light intensity in a controlled-environment chamber. **a**, Light response curves of assimilation of plants measured at 400 ppm and 2,000 ppm CO_2_ concentration, and stimulated respiration during the first minutes after the end of illumination with saturating actinic light. **b**, Photosynthetic parameters, measured only at 400 ppm CO_2_ concentration. Nt-JF1006-30 served as the TALEN control harbouring the unaltered *nad9* allele. NPQ, non-photochemical quenching^69^; qL, measure of the redox state of the photosystem II (PSII) acceptor side^70^; Y(NO), measure of the non-regulated thermal dissipation of excitation energy, an indicator of PSII photoinhibition (if increased); Y(ND), measure of the donor side limitation of PSI by linear electron transport; Y(NA), measure of the acceptor side limitation of PSI by the Calvin–Benson cycle and other downstream metabolic reactions^71^. For the wild type and the TALEN-control line Nt-JF1006-30, *n* = 6 biological replicates (independent plants) were measured. For the single mutant E94K-1 and the double mutant D93E/E94K-1, *n* = 5 independent plants (biological replicates) were measured, except for the assimilation measurements at 2,000 ppm CO_2_ for the single mutant E94K-1, where *n* = 4 biological replicates were analysed. The youngest fully expanded leaves were analysed. The average values and standard deviations are shown. Where the error bars are not visible, the standard deviation was smaller than the size of the symbol.

**Extended Data Fig. 9:**
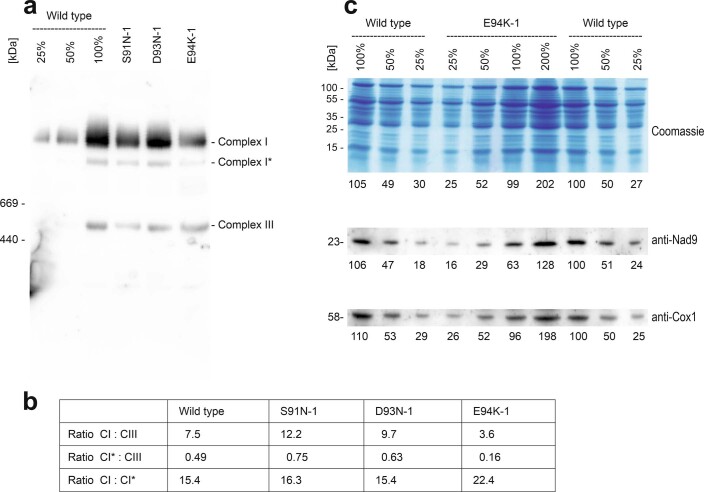
Characterization of complex I in a set of selected mitochondrial *nad9* mutants. (**a**) Analysis of complex I accumulation by BN-PAGE and immunoblotting. A replicate gel of the gel shown in Fig. 4a was blotted and hybridized to an anti-CA2 antibody recognizing one of the carbonic anhydrase subunits of complex I. One of two technical replicates with similar results is shown. (**b**) Quantification of the ECL signal intensities in the immunoblot shown in panel (a). (**c**) Quantification of Nad9 protein amounts in the *nad9* E94K-1 mutant after SDS-PAGE analysis (in a 12% polyacrylamide gel) and immunoblotting. The percentages above the Coomassie-stained gel indicate the relative protein amounts loaded in each lane. The numbers below each panel represent the relative staining intensities (upper panel) and the intensities of the hybridization signals (middle and bottom panels). Quantification was done with the Fiji image processing package. The 100% wild-type sample in lane 8 served as reference. For information about the total number of SDS-PAGE and anti-Nad9 western blot experiments, see Fig. 4. The anti-Cox1 western blot was performed once. Quantifications were done based on the images shown here. Source data

Selected lines were subjected to detailed physiological and biochemical analyses (Figs. 4 and 5 and Extended Data Fig. 9). Measurements of gas exchange rates of plants grown at 350 µE m^−2^ s^−1^ light intensity in a controlled-environment chamber revealed no detectable defects in mitochondrial respiration (Fig. 5a). Because defects in complex I function are known to also affect photosynthesis^53^, photosynthetic parameters were determined in wild-type and mutant plants. These measurements provided no evidence of impaired electron transfer or photosynthetic carbon fixation (Fig. 5a,b).

**Extended Data Fig. 10:**
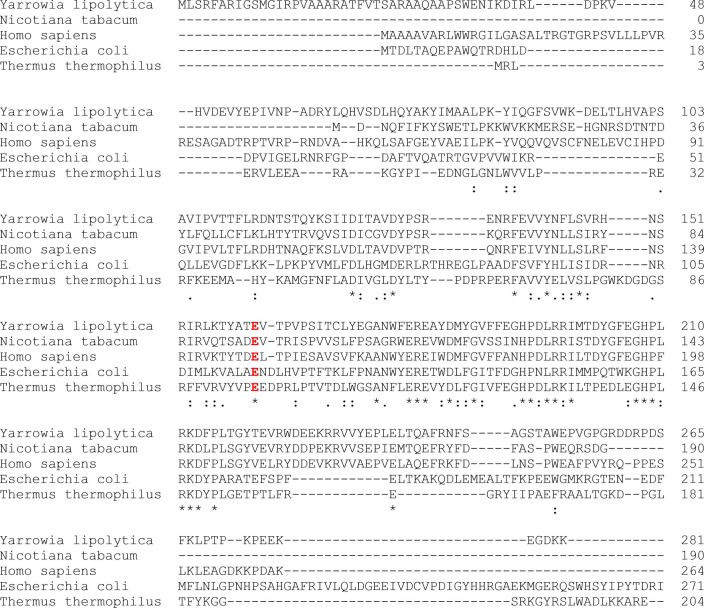
Amino acid sequence alignment of the Nad9 protein from tobacco with homologues from different species of prokaryotes and eukaryotes. The highly conserved glutamate residue 94 is highlighted in red. Asterisks (*) indicate perfect conservation, colons (:) denote residues belonging to a group of amino acids exhibiting strong similarity, and dots (.) mark residues belonging to a group of amino acids exhibiting weak similarity. For the tobacco Nad9 protein, the (unedited) genomic sequence was used.

To specifically examine complex I accumulation and activity, we also analysed mitochondrial protein complexes in three selected mutants (S91N, D93N and E94K) by native gel electrophoresis and immunoblotting (Fig. 4 and Extended Data Fig. 9). While two of the mutants (S91N and D93N) showed no pronounced alterations compared with the wild type, the E94K mutant plants displayed reduced complex I accumulation (Fig. 4a and Extended Data Fig. 9). The functional importance of E94 is in line with the high evolutionary conservation of this position in prokaryotes and eukaryotes (Extended Data Fig. 10). Immunodetection of Nad9 with specific antibodies revealed lower protein accumulation levels in E94K plants (approximately 60% of wild-type levels; Extended Data Fig. 9), suggesting reduced stability of the mutant Nad9 protein. Interestingly, the Nad9 protein of the E94K mutant also displayed altered electrophoretic mobility (Fig. 4b), thus providing direct biochemical evidence of the relevance of the mutation at the protein level.

## Discussion

Due to their independence of RNA molecules, TALEs currently provide the method of choice for mitochondrial genome editing^54^. In previous studies, TALENs have been shown to induce large deletions in the mitochondrial genome of plants, presumably due to double-strand break repair by microhomology-mediated illegitimate recombination^29,55^. The difficulties in predicting the breakpoints of such deletions and the risk of the deletion spanning multiple genes or including essential mitochondrial genes limit the usefulness of the method in reverse genetics and mitochondrial genome engineering for biotechnology. Base editing technologies^20–22,56^ potentially provide more precise and versatile methods for the targeted introduction of genetic changes into mitochondrial genomes. A recent study demonstrated the possibility to introduce C-to-T mutations into human mtDNA with a bacterial cytidine deaminase fused to TALEs^30^. Preliminary evidence suggests that the method can also be applied to plants, although homochondriomic mitochondrial mutants that are fertile and transmit the mutated mitochondrial genomes into the next generation have not been reported^31^. Moreover, the TALE–cytidine deaminase fusion has several serious limitations, including its restriction to a single mutation type (C-to-T transitions) and a pronounced sequence context preference^30^ that makes most positions in the mitochondrial genome inaccessible to editing.

In the course of this work, we have developed a method that addresses these limitations. TALEN-GDM relies on site-specific cleavage of the mitochondrial genome and the assumption that the resulting double-strand breaks cannot be fixed by religation but rather are repaired by homologous recombination (using an uncut genome as the repair template^40^) or, in rare cases, by microhomology-mediated illegitimate recombination^29^. Since recombination repair involves DNA polymerization, repair errors by nucleotide misincorporation can occur that lower the sensitivity of the target sequence to TALEN-induced cleavage and, in this way, can set off a gene-drive-like selection effect. We have shown that the application of the DNA-intercalating agent EtBr accelerates the appearance of such mutations, thus enhancing the efficiency of TALEN-GDM (Table 1 and Supplementary Table 1).

Applying TALEN-GDM, we successfully isolated a large set of homochondriomic mutants in the mitochondrial *nad9* gene of tobacco (Fig. 3b and Supplementary Table 1). The mutants stably transmitted their genetic changes to the progeny in the expected non-Mendelian (uniparentally maternal) fashion. The frequency of mitochondrial mutant recovery was high: with the EtBr protocol, more than 5% of the sequenced regenerants carried a mutation in the target sequence (Table 1 and Fig. 3b). This frequency makes TALEN-GDM a workable method for the efficient introduction of mutations into the plant mitochondrial genome.

In this work, we identified mitochondrial mutants by sequencing of individual regenerated shoots (Fig. 2). However, in view of the fast enrichment of the mutated mitochondrial genome (Fig. 3a), it would be straightforward to set up high-throughput strategies based on sequencing of pooled regenerants, which would greatly increase the power of the method and facilitate the large-scale identification of nearly unlimited numbers of mutations in the target sequence.

Importantly, our TALEN-GDM method does not suffer from the limitations that base editors have with respect to mutation types and sequence context requirements. We have recovered a wide spectrum of different transitions and transversions, and mutations in many different positions of the TALEN binding site (Fig. 3b). These findings suggest that TALEN-GDM provides a relatively unbiased method of site-directed mutagenesis. This feature should make TALEN-GDM a useful tool for the analysis of mitochondrial gene functions by reverse genetics, in that it allows the recovery of viable homochondriomic mutants even for essential mitochondrial genes. Unbiased mutagenesis in a target sequence covering six to seven codons (Fig. 3b) greatly increases the chances of obtaining useful hypomorphic mutants (in which the mutated gene product possesses a reduced level of activity), similar to our E94K mutant that reduces Nad9 protein accumulation to ~60% of the wild-type level.

The TALEN-GDM method developed in the course of this work will be applicable to all species in which nuclear transformation is available. Thus, TALEN-GDM may also become a valuable tool in applied research aiming at the engineering of agronomically valuable traits into plant mitochondrial genomes. Most importantly, TALEN-GDM may facilitate the identification of novel mitochondrial mutations that confer cytoplasmic male sterility, a trait that is of paramount importance in plant breeding^57–59^. Finally, the TALEN-GDM method will be applicable to all organisms that are recalcitrant to direct transformation of their organellar genomes, including the mitochondria of humans and other animals, and the plastids of cereals.

## Methods

### Construction of TALEN vectors

The TALEN sequences were designed according to established principles. Briefly, the TALEN effector binding elements are 18 bp long, preceded by a thymine and separated by a 12-bp spacer sequence. Suitable target sequences in *nad9* were identified by eye, preferring cytosines and avoiding guanines, because the C-binding repeats have high affinity and specificity, whereas the G-binding repeats have relatively low affinity and specificity. Stretches of identical bases were also avoided. The selection of suitable TALEN effector binding elements was additionally based on the presence of restriction endonuclease recognition sites (for example, BsrGI) within the spacer sequence (that is, the sequence between the two TALEN binding sites).

For the construction of the TALEN vectors, TALE-encoding modules lacking the repeats were assembled with BsaI site-flanked modules encoding the short CaMV 35S promoter (from vector pICH51277; ref. ^60^), a mitochondrial presequence (AtIVD; ref. ^61^) with a triple HA-tag (forward TALEN; ‘left’ TALEN) or a triple FLAG-tag (reverse TALEN; ‘right’ TALEN), the truncated TALE N terminus and C terminus, the FokI endonuclease^62^, and the *OCS* terminator from *Agrobacterium tumefaciens* (from plasmid pICH41432; ref. ^60^) into vectors pICH47732 and pICH47742 (ref. ^60^), yielding vectors pICH47732–TALEN∆Rep and pICH47742–TALEN∆Rep, respectively. The repeat domains of the TALENs were created using a previously described method^63^ and cloned via BpiI sites into pICH47732–TALEN∆Rep and pICH47742–TALEN∆Rep. TALEN modules with repeats were assembled together with pICH47751–Kanamycin, conferring in planta resistance to kanamycin, and pICH47766 into pICH50505 (ref. ^60^) using the BpiI restriction sites. The plasmid sequences of the final plant transformation vectors pJF1004, pJF1005 and pJF1006 were deposited in GenBank under accession numbers MZ417370, MZ417371 and MZ417372. In all constructs, the repeat variable diresidues NI (for A), HD (for C), NN (for G) and NG (for T) were used.

### Plant material and growth conditions

*Nicotiana tabacum* cultivar Petit Havana was used for all experiments. The plants were raised from seeds under aseptic conditions on synthetic medium (MSsuc3 medium). The medium consisted of Murashige and Skoog (MS) salts^64^ supplemented with modified MS vitamins (Duchefa M0245) and 3% sucrose. The pH was adjusted to 5.8, and the medium was solidified with 0.68% agar (Duchefa M1002). Calli and shoots were induced on medium NtSIM1, which is MSsuc3 medium supplemented with 0.1 g l^−1^ 1-naphthaleneacetic acid and 1 mg l^−1^ 6-benzylaminopurine. Rooting of regenerated shoots was induced in MSsuc3 medium.

If not stated otherwise, the photoperiod was 16 h of daylight (light intensity 25–50 µE m^−2^ s^−1^) at 25 °C followed by 8 h of darkness at 20 °C. The seeds were surface-sterilized in a desiccator with chlorine gas (released by adding 3% v/v of 37% HCl to a 13% solution of NaOCl) for 5 h or by incubation in 70% ethanol (+0.3% Tween20) followed by incubation in 6% NaOCl (+0.3% Tween20) for 2 min each. In the greenhouse, the plants were grown in soil under standard conditions (16 h day length; temperature regime, 25 °C during the day and 20 °C at night; average light intensity, 300 µE m^−2^ s^−1^). For some growth analyses and physiological measurements, plants were grown in a controlled-environment chamber under long-day conditions (16 h light) at 22 °C and 75% relative humidity during the day, and 18 °C and 60% relative humidity during the night. For the high-light growth experiments, the light intensity was set to 1,000 µE m^−2^ s^−1^. For the gas exchange and photosynthetic analyses, plants were grown at 350 µE m^−2^ s^−1^ light intensity.

### Plant transformation and characterization of transgenic lines

*Agrobacterium tumefaciens* strain GV2260 was used for the plant transformation experiments. Transgenic lines were selected on an MS-based regeneration medium supplemented with 50 µg ml^−1^ kanamycin.

To identify plants that functionally express the TALEN transgenes (that is, display cleavage of the mitochondrial *nad9* locus), total DNA was isolated by a cetyltrimethylammoniumbromide-based method^65^ from T_1_ plants and subjected to restriction fragment length polymorphism analysis by Southern blotting. To this end, DNA samples were digested with restriction enzymes, separated in 1% or 2% agarose gels by electrophoresis and blotted onto Hybond-XL membranes (GE Healthcare). Primers oJF271 and oJF272 (Supplementary Table 2) were used to produce an *nad9*-specific hybridization probe by PCR amplification. The probe was purified with the NucleoSpin Gel and PCR Clean‑up kit (Macherey-Nagel) and labelled with α-[^32^P]dCTP by random priming (GE Healthcare) according to the manufacturer’s instructions. Hybridizations were performed at 65 °C in Church buffer (1% BSA, 1 mM EDTA, 7% SDS, 500 mM Na_2_HPO_4_, pH 7.2) overnight. Signals were detected with a Typhoon Scanner (GE Healthcare). The *nad9* cleavage efficiency was quantified using the Fiji (ImageJ) image processing package by calculating the amount of signal in the two TALEN-cut bands (712 bp and 436 bp) as a percentage of the total *nad9* signal (the uncut 1,148-bp band plus the two TALEN-cut bands).

The *nad9* sequences in the T_0_ plants were screened for mutations by PCR amplification with primers oJF271 and oJF272, followed by bulk Sanger sequencing of the amplification products (LGC Genomics). Template DNA was extracted with Extraction Solution Molecular Biology (Sigma-Aldrich). To address the possibility that plants might be mosaic and/or heterochondriomic, DNA for PCR analyses was always extracted from a pool of material harvested from three consecutive leaves (to likely cover offspring of all stem cells in all layers).

The presence of the genes encoding each TALEN arm in the TALEN-active lines was analysed by PCR with primers oJF1147 and oJF1323 (Supplementary Table 2). β-TUBULIN as a control locus was amplified with primers oJF1028 and oJF1029. The DNA size marker shown in all PCR gel images is the GeneRuler 100 bp Plus DNA Ladder (Thermo Scientific). RT–PCR with cDNA synthesized from total RNA with primers oJF1026 (β-TUBULIN) and oJF1300 (TALENs) was performed to assess the expression of the HA–TALEN and FLAG–TALEN arms using primers oJF1027 and oJF748 (for β-TUBULIN as an internal control), oJF1298 and oJF748 (HA-tag specific), oJF1299 and oJF748 (FLAG-tag specific), and oJF1147 and oJF748 (for the amplification of both HA-tag and FLAG-tag).

To verify that the double-strand break in *nad9* detected in our Southern blot analyses is indeed caused by TALEN cuts, total DNA was blunted using mung bean exonuclease (NEB) and ligated to a short synthetic double-stranded DNA fragment created by annealing oligonucleotides oJF650 and oJF651 (Supplementary Table 2). Ligation products with *nad9* were amplified using an adapter primer (oJF060) combined with the following gene-specific primers: oJF271 (upstream 1), oJF317 (upstream 2), oJF272 (downstream 1) and oJF499 (downstream 2; Supplementary Table 2 and Extended Data Fig. 3).

### Identification of mitochondrial mutants

To isolate mitochondrial mutants, verified TALEN-expressing lines were raised from seeds under aseptic conditions, and the leaves were cut into pieces and placed onto shoot induction medium (NtSIM1; see above). The shoots were transferred to Magenta boxes with rooting medium (MSsuc3; see above) and genotyped. If no mutation in *nad9* was found, a new regeneration round was initiated with leaf pieces from these plants. This process was repeated up to ten times. If a mutation was found, an additional regeneration round was conducted to reduce actual or apparent heterochondriomy. After rooting, confirmed homochondriomic plants were transferred to the greenhouse for seed production by selfing or fertilization with wild-type pollen (to remove possible tissue-culture-induced somaclonal variation from the nuclear genome). Descendants of this backcross were used for phenotyping.

For experiments with mutagens, the mutagenic chemicals were either added to the culture medium or used for seed imbibition or treatment of leaf pieces. EtBr was applied as an aqueous solution at concentrations of 0.2% for seed imbibition and 0.0002% to 0.002% in the culture medium. NEU was dissolved in a mixture of 70% ethanol and 0.1% acetic acid and used at a final concentration of 5 mM.

To investigate the apparent heterochondriomy caused by the presence of promiscuous mtDNA in the nuclear genome, genotyping was performed with DNA extracted from purified mitochondria (see below). In addition, total RNA was isolated from mutant plants with the NucleoSpin RNA Plant kit (Macherey-Nagel) and reverse transcribed with SuperScript III Reverse Transcriptase (Invitrogen) using the *nad9*-specific primer oJF1113 (Supplementary Table 2). Full-length *nad9* cDNA was then amplified with primers oJF311 and oJF748 (Supplementary Table 2), and the RT–PCR products were purified and bulk sequenced as described above. In addition to the homochondriomic versus heterochondriomic status of the mutation of interest, the cDNA sequences were analysed for possible aberrations in mRNA editing patterns and the possible presence of additional (off-target) mutations elsewhere in the coding sequence of *nad9*. For amplicon deep sequencing, total RNA isolated from kanamycin-resistant seedlings was reverse transcribed with primer oJF1302 and used as a template for PCR amplification with primers oJF1304 and oJF1301 (Supplementary Table 2).

### Isolation of mitochondria

Mitochondria were purified from 40 g of fresh leaf material. The leaves were homogenized in extraction buffer (300 mM sucrose, 15 mM potassium pyrophosphate, 2 mM EDTA, 10 mM KH_2_PO_4_, 1% (w/v) PVP-40, 1% (w/v) BSA, 20 mM sodium ascorbate, 5 mM cysteine, pH 7.5) in a Waring blender. The homogenate was filtered through two layers of Miracloth and then centrifuged at 2,000 *g*. The supernatant was collected and centrifuged at 20,000 *g*, and the pellet was resuspended in wash buffer (300 mM sucrose, 1 mM EGTA, 10 mM MOPS–KOH, pH 7.2). Following centrifugation at 2,000 *g*, the supernatant was collected and centrifuged at 20,000 *g*, and the pellet obtained was resuspended in a small volume of wash buffer and then loaded onto a Percoll step gradient consisting of one volume of 50% (v/v) Percoll, five volumes of 25% (v/v) Percoll and one volume of 18% (v/v) Percoll (all solutions were prepared in gradient buffer: 300 mM sucrose, 10 mM MOPS–KOH, pH 7.2). The gradient was centrifuged at 40,000 *g* for 45 min, and the mitochondria were collected from the interface between the 50% and 25% solutions and then washed several times with wash buffer. Protein concentration was estimated using the Bradford method (ROTI-Quant, Carl Roth).

### Protein extraction and immunoblot analysis

Samples of 20 µg of extracted mitochondrial protein were solubilized in loading buffer (80 mM Tris–HCl, pH 6.8, 25 mM EDTA, 0.1 M DTT, 1% (v/v) glycerol, 2% (w/v) SDS, 0.05% (w/v) bromophenol blue) and separated by electrophoresis in 12% SDS–PAA gels. The proteins were then transferred to PVDF membranes (Immobilon-P, Merck Millipore) in a tank blotter (transfer buffer: 192 mM glycine, 25 mM Tris, pH 8.3). Equal loading and successful transfer were confirmed by Coomassie staining of the membrane. The blotted proteins were then immunodecorated with specific antibodies, and the signals were detected by chemiluminescence with secondary horseradish peroxidase-conjugated antibodies (ECL Prime, GE Healthcare). Images were acquired using the Fusion-FX system (Vilber Lourmat). Polyclonal antibodies against Nad9 (ref. ^66^) were used at a dilution of 1:20,000, and polyclonal antibodies against Cox1 (ref. ^67^) were used at a dilution of 1:10,000. Anti-rabbit-HRP conjugate from Sigma (A0545) was used as a secondary antibody at a dilution of 1:10,000.

### BN–PAGE and NADH oxidase staining

For the analysis of mitochondrial membrane protein complexes via BN–PAGE, mitochondria equivalent to 200 µg of protein were solubilized with 5% (w/v) digitonin (solubilization buffer: 150 mM potassium acetate, 10% (v/v) glycerol, 30 mM HEPES, pH 7.4) and loaded on a 4.5–16% gradient BN–PAA gel. Following electrophoretic separation of protein complexes, the gel was stained with colloidal Coomassie. For NADH oxidase staining, the gel was washed twice in water and then incubated in staining solution (0.14 mM NADH, 1 mg ml^−1^ nitroblue tetrazolium, 100 mM Tris–HCl, pH 7.4). When satisfactory staining intensity had been reached, the reaction was stopped with fixing solution (50% (v/v) methanol, 10% (v/v) acetic acid). Stained gels were scanned using a flatbed scanner. Protein complexes from replicate gels were transferred to PVDF membranes (Immobilon-P) by tank blotting (transfer buffer: 192 mM glycine, 25 mM Tris, pH 8.3). Equal loading and successful protein transfer were confirmed by Coomassie staining of the membranes. Proteins were labelled with specific antibodies and detected by chemiluminescence as described above. Polyclonal antibodies against the CA2 subunit of complex I (ref. ^68^) were used at a dilution of 1:10,000.

### Measurements of gas exchange and photosynthetic parameters

Gas exchange measurements were performed with a GFS-3000 gas exchange system equipped with the LED array unit 3056-FL as an actinic light source (Heinz Walz). Light response curves of CO_2_ assimilation were recorded at 22 °C cuvette temperature and 17,500 ppm humidity. Measurements were performed at two CO_2_ concentrations, 400 ppm and 2,000 ppm (a saturating CO_2_ concentration), to fully repress photorespiration and determine the capacity of photosynthesis. The youngest fully expanded leaves of plants grown at 350 µE m^−2^ s^−1^ light intensity were used for the measurements. At this developmental state, tobacco leaves have their highest capacity of both respiration and assimilation. After leaf respiration had been determined in darkness, the actinic light intensity was increased stepwise to 100, 350, 750 and finally 1,500 µE m^−2^ s^−1^. At each light intensity, gas exchange was recorded until the steady state of leaf assimilation had been reached. After the final saturating illumination step at 1,500 µE m^−2^ s^−1^, the actinic light was switched off, and respiration was recorded until a constant respiration rate in darkness had been reached.

Parameters of photosynthetic electron transport were determined with the Modular version of the Dual-PAM-100 instrument (Heinz Walz) at 22 °C. Light response curves of chlorophyll *a* fluorescence and PSI parameters were measured after 20 min of dark adaptation, to first determine the maximum quantum efficiency of PSII in the dark-adapted state (*F*_V_/*F*_M_). Afterwards, light response curves of linear electron transport, the chlorophyll *a* fluorescence parameters non-photochemical quenching (qN; ref. ^69^) and qL, a measure of the redox state of the PSII acceptor side^70^, and measures of the acceptor-side (Y(NA)) and donor-side limitation of PSI (Y(ND)) were determined^71^. The measuring times at each actinic light intensity were 150 s under light-limited conditions and 60 s under light-saturated conditions.

Linear electron transport was corrected for leaf absorptance, which was calculated as 100% incident light minus light transmitted through the leaf (%) minus light reflected on the leaf surface (%). Transmittance and reflectance spectra between 400 and 700 nm wavelength were recorded using an integrating sphere attached to a photometer (V650, Jasco). The spectral bandwidth was set to 1 nm, and the scanning speed was 200 nm min^−1^. Measurements of chlorophyll content and the chlorophyll *a*/*b* ratio were done with a Jasco V-630 photometer (Jasco) in 80% (v/v) acetone^72^.

### DNA sequence analyses and artwork

The Lasergene suite (DNASTAR) and the SnapGene Viewer (https://www.snapgene.com/snapgene-viewer/) were used for the DNA sequence analyses. Figure 2a and Extended Data Fig. 1 were created with pre-drawn icons from BioRender (https://biorender.com).

### NGS

NGS was performed at the Sequencing Core Facility of the Max Planck Institute for Molecular Genetics. For NGS analysis of mitochondrial genomes (mtDNA-seq), DNA was extracted from an aliquot of the mitochondria isolated for protein analysis. After initial quality control with the Bioanalyzer (Agilent), sequencing libraries were prepared from 30 ng of DNA per sample following the KAPA DNA HyperPrep Kit (Roche) library preparation protocol for double-indexed Illumina libraries. Briefly, DNA was sheared using a Covaris S2 system (duty cycle 5%, intensity 5, 40 s run time). After end repair and A-tailing, Illumina-sequencing-compatible adapters carrying unique dual indices were ligated (NEXTFLEX Unique Dual Index Barcodes). Following bead-based clean-up steps, the libraries were amplified using seven cycles of PCR. Library quality and size were checked with qBit, the Bioanalyzer and quantitative PCR. Sequencing was carried out on an Illumina MiSeq Nano flow cell in PE150bp mode, yielding between 240,000 and 260,000 sequenced fragments per sample. The read coverage across the mitochondrial genome is displayed in Extended Data Fig. 10.

For NGS amplicon sequencing (amplicon-seq), RT–PCR products were purified using the NucleoSpin Gel and PCR Clean‑up kit (Macherey-Nagel). After initial quality control with the Bioanalyzer, sequencing libraries were prepared from 50 ng of DNA per sample according to the KAPA DNA HyperPrep Kit (Roche) library preparation protocol for double-indexed Illumina libraries. After end repair and A-tailing, Illumina-sequencing-compatible adapters carrying unique dual indices were ligated (NEXTFLEX Unique Dual Index Barcodes). Following bead-based clean-up steps, the libraries were amplified using five cycles of PCR, and library quality and size were checked with qBit, the Bioanalyzer and quantitative PCR. Sequencing was carried out on an Illumina MiSeq Micro flow cell in PE150bp mode, yielding between 140,000 and 200,000 sequenced fragments per sample.

### Bioinformatic analyses of NGS data

An initial quality check of the sequence data of all samples from both NGS datasets (mtDNA-seq and amplicon-seq) was performed with FastQC v.0.11.9 (https://www.bioinformatics.babraham.ac.uk/projects/fastqc/). Three additional pre-mapping steps were conducted for the amplicon-seq data. First, read pairs were joined using PEAR (ref. ^73^) v.0.9.11 (-v 65) to avoid doubled coverage counting and increase the quality of the overlapping part of the pairs, in which the introduced mutations are located. Second, Flexbar (ref. ^74^) v.3.5 was used to remove the additional amplicon-specific sequence (CGTCACGAACGTACTTTGGA; introduced during the cDNA synthesis step as a 5′ extension of primer oJF1302) with the adapted options -at ANY -m 213. Third, reads representing the reference DNA allele (C) instead of the known RNA-edited sequence (T) within the sequences GTTT(C/T)GAT, GGC(C/T)GGTG and TCT(C/T)CGGT were considered to originate from contaminating DNA and removed.

FASTQ files of both datasets were then mapped against the NCBI NC_006581.1 reference with BWA v.0.7.17 in MEM mode^75^. Sample-wise alignment files were merged dataset-wise with SAMtools (ref. ^76^) v.1.12. SNPs were afterwards called with Freebayes (ref. ^77^) v.1.3.2 with slightly adapted options (-p 1 -n 2) and annotated with SnpEff (ref. ^78^) v.4.3k. The database was built from the GenBank file of NC_006581.1 using the genbank build option in SnpEff. To confirm homochondriomy, the alignment files of the amplicon-seq dataset were further processed with SAMtools mpileup (-d 200000 -Q 40), and nucleotide-wise coverage was extracted with a user-defined Perl script for all positions at which SNPs were introduced and four additional randomly chosen positions as non-SNP control sites.

### Reporting Summary

Further information on research design is available in the Nature Research Reporting Summary linked to this article.

## Supplementary information

Supplementary Information Supplementary Methods and Tables 1 and 2.

Reporting Summary

Supplementary Data Supplementary Data 1: Results from NGS of RT–PCR amplicons from 18 *nad9* mutant lines. For assessment of homochondriomy at the *nad9* target site, RT–PCR products were generated from pools of seedlings (selected for kanamycin resistance) with cDNA first-strand synthesis primer oJF1302 and PCR primers oJF1301 and oJF1304, and analysed by deep sequencing. Only PCR products displaying RNA editing were used for analysis to exclude contaminating promiscuous *nad9* sequences in the nuclear genome. Nucleotide distributions at the positions of interest and four randomly chosen control positions (not mutated in any of the 18 lines listed) are shown. The lines mutated at the respective positions are highlighted in grey. Nucleotides corresponding to the respective wild-type allele are found only at background levels (that is, in less than 0.1% of the reads) in the mutant lines, strongly suggesting homochondriomy. Supplementary Data 2: Results from NGS of whole mtDNA of the mutant lines D93N-1, E94K-1 and S91N-1. The upper table lists all positions in which deviations from the reference genome NC_006581 (from cultivar Bright Yellow 4) were detected. The positions with the expected *nad9* mutations are marked in green. All other 12 deviating positions are due to sequence polymorphisms between cultivar Petit Havana (used in this work) and cultivar Bright Yellow 4 (used to determine the reference genome sequence), as demonstrated by amplification of total DNA from Petit Havana and Sanger sequencing of the regions containing the polymorphisms with the primer pairs listed in the bottom table. Thus, no off-target mutations were induced in the mitochondrial genomes by TALEN-GDM. The genome sequencing also provides additional confirmation of homochondriomy of the induced point mutations.

## Data Availability

The data supporting the findings of this study are available within the paper and its Supplementary Information files. The NGS datasets were deposited under BioProject ID PRJNA787054 (https://www.ncbi.nlm.nih.gov/bioproject/PRJNA787054). The sequences of plasmids pJF1004, pJF1005 and pJF1006 are accessible via GenBank accession numbers MZ417370, MZ417371 and MZ417372. GenBank accession number NC_006581.1 was used as the tobacco mitochondrial reference genome. Source data are provided with this paper.

## Source data

Source Data Extended Data Fig. 9 Unprocessed western blots.

## Code availability

The Perl code (extract_BaseCovInformation.pl) employed is available in the GitHub repository at https://github.com/AxelMacFoly/ngs_perl_scripts/.
