# Supplementary material for: Targeted introduction of heritable point mutations into the plant mitochondrial genome
Source: Nat Plants. 2022 Mar 17;8(3):245–56. doi: 10.1038/s41477-022-01108-y (PMC8940627; doi:10.1038/s41477-022-01108-y)
Supplement: Supplementary file 1 — Supplementary Methods and Tables 1 and 2. [file 41477_2022_1108_MOESM1_ESM.pdf]

nature portfolio

https://doi.org/10.1038/s41477-022-01108-y

Supplementary information

Targeted introduction of heritable point mutations into the plant mitochondrial genome

In the format provided by the authors and unedited

Supplementary Methods

Short protocol and timeline for the optimized procedure to obtain mitochondrial point mutants by TALEN-GDM

1. Transform the nuclear genome of tobacco (*N. tabacum*) with the construct encoding mitochondrially targeted TALENs using standard protocols for *Agrobacterium tumefaciens*-mediated transformation.
2. Isolate transgenic lines (T_0 plants) by suitable selection and regeneration protocols.
3. Screen T_0 plants (or their T_1 offspring) for TALEN cleavage activity by testing for DNA cuts at the TALEN target sites by Southern blotting.
4. Maintain lines with active TALENs by plant propagation via selfing (or cross-fertilization with wild-type pollen).
5. Germinate T_1 seeds from TALEN-active lines in sterile culture with the appropriate antibiotic to ensure presence of the TALEN transgene.
6. Prick out individual resistant seedlings, and transfer them to sterile Magenta boxes with synthetic medium (after approximately 2-3 weeks).
7. Harvest young leaves and cut them into pieces of approximately 4 mm x 4 mm (4-6 weeks after pricking out). Transfer the explants to shoot induction medium supplemented with 0.002% (w/v) ethidium bromide.
8. When shoots appear (after 4-8 weeks), transfer individual shoots to Magenta boxes with synthetic medium (without ethidium bromide). Harvest tissue samples from the three youngest leaves for genotyping (by PCR and DNA sequencing).
9. If genotyping does not reveal any mutation at the target site, take new leaves from the transferred shoots from step (8), and start again at step (7). If genotyping reveals mutations at the target locus, continue with step (10).
10. To achieve homochondriomy, take at least three consecutive new leaves from the transferred shoots from step (8), cut them into pieces of approximately 4 mm x 4 mm, and place the explants onto the surface of shoot induction medium (not supplemented with ethidium bromide).
11. When shoots start appearing (after approximately 4 weeks), take individual shoots and transfer them to boxes with synthetic medium (without ethidium bromide). Harvest material from the three youngest leaves for genotyping by PCR and DNA sequencing. To avoid wild-type signals from promiscuous mitochondrial DNA sequences in the nuclear genome, isolate mitochondrial DNA or use cDNA. If cDNA is used, amplify and sequence a fragment that ideally contains at least one RNA editing site.
12. If genotyping reveals continued heterochondriomy of the mutation at the target site, take new leaves from the transferred shoots from step (11) and start again at step

(10). If genotyping reveals homochondriomy of the mutation at the target locus, continue with step (13).

13. Let the shoots from step (11) that tested positive for homochondriomy in step (12) root and continue to grow until the plantlets can be transferred to soil and grown in the greenhouse. Upon flowering, fertilize the plants with wild-type pollen to (i) reduce the (epi-)genetic burden imposed by tissue culture, and (ii) facilitate outcrossing of the nuclear transgenes (including the TALEN genes).
14. Raise the next generation from seeds harvested after step (13). Grow plants to maturity under standard greenhouse conditions. Reconfirm presence and homochondriomy of the desired mitochondrial mutation, and verify absence of TALEN transgenes by genotyping via PCR. Fertilize plants with wild-type pollen, and harvest seeds. If the mitochondrial mutation is confirmed, TALEN transgenes are absent, and enough seeds have been obtained, the procedure is complete. If TALEN transgene(s) are still present in all progeny tested, continue with step (15).
15. Grow plants from seeds harvested in step (14) in the greenhouse, check for presence and homochondriomy of the desired mitochondrial mutation, and test for absence of TALEN transgenes by (PCR-based) genotyping. Select plants with the mitochondrial mutation confirmed and absence of TALEN transgenes verified. Fertilize again with wild-type pollen. The procedure is complete after seed harvest.

Remark: Always work with as many lines and/or individual plants in parallel as possible. It is recommended to test several dozen lines (regenerated shoots) in steps (2) and (11), and at least a hundred shoots in step (8).

See also Fig. 2 and Extended Data Fig. 1.

Supplementary Table 1. Overview of mitochondrial mutants obtained from TALEN-GDM of *nad9*.

Nucleotide exchange	Amino acid exchange	Line names
G254T	R85L	R85L-1[a]
C265A	Q89K	Q89K-1[a] and Q89K-2[b]
C269A	T90N	T90N-1[a] (lost; line could not be recovered)
A271G	S91G	S91G-1[c] and S91G-2[a]
G272A	S91N	S91N-1[d] and S91N-2[e]
T273C	S91S	S91S-1[f]
A276G	A92A	A92A-1[g] and A92A-2[h]
G277A	D93N	D93N-1[f], D93N-2[i], D93N-3[j]
A278G	D93G	D93G-1[a]
C279A/G280A	D93E/E94K	D93E/E94K-1[a]
G280A	E94K	E94K-1[f] and E94K-2[e] (line 2 died)
G283A	V95I	V95I-1[a]
T284A	V95E	V95E-1[b]

[a] from experiment described in Table 1

[b] constant light and 0.001% ethidium bromide (EtBr); obtained after 1st regeneration round (Rr); seeds not treated with mutagen

[c] no mutagen employed; 16 h light / 8 h dark; mutation detected in 6th Rr

[d] seeds incubated in 5 mM N-nitroso-N-ethylurea (NEU) for 2 h before sowing, leaves from seedlings cut and incubated in 5 mM NEU before exposure to regeneration medium without mutagen for 1st Rr; 1 out of 5 shoots analyzed had a mutation

[e] leaves from 5 shoots obtained in [d] incubated in 5 mM NEU and regenerated on medium without mutagen (2nd Rr); line S91N-2 possibly clonally derived from line S91N-1

[f] seeds incubated in 0.2% EtBr at 4°C for 40-44 h before sowing, leaves from seedlings cut and incubated in 5 mM NEU prior to regeneration on medium without mutagen; mutations detected after 2nd Rr

[g] seeds incubated in 0.2% EtBr at 4°C for 40-44 h before sowing, 1st Rr on medium without mutagen, 2nd Rr on 0.001% EtBr; mutation found in all shoots of 2nd Rr (i.e., likely already present in 1st Rr)

[h] from a supertransformation experiment with the TALEN line; mutation detected in T_1 plants

[i] seeds incubated in 5 mM NEU for 2 h before sowing, 1st Rr on medium without mutagen, 2nd Rr on 0.0002% EtBr; mutation detected after 2nd Rr

[j] line obtained from [e]; not further propagated (due to redundancy with lines D93N-1 and -2)

[d,e,f,g,h,j] 16 h day length until 1st Rr; constant light in 2nd Rr

Supplementary Table 2. List of oligonucleotides used in this study.

Name	Sequence (5' → 3')	Target
oJF060	GTTGTGTGGAATTGTGAGC	adapter
oJF271	AACAGGTACCGTTGGGAGACTTTACCCAAG	*nad9*
oJF272	TGTTGGATCCTTATCCGTCGCTACGCTGT	*nad9*
oJF311	CCAAGCAATGCCCAAAAGTCCCATGC	*nad9* (upstream)
oJF317	AACAGGTCTCAGACTAAATTTCAATTGTTGTGCTTTCTG	*nad9*
oJF496	GAGTTGATTATCCCTCTCGA	*nad9*
oJF499	TCTTGGGTCATCTCAATGGG	*nad9*
oJF650*	AGAGCTCACAATTCCACACAACGAAC	adapter
oJF651	GTTGTGTGGAATTGTGAGCTCT	adapter
oJF748	ATGCCGGACATCTTAAGGAG	adapter
oJF1026	ATGCCGGACATCTTAAGGAGTCTGTACTGCTGAGAACCAC	β-TUBULIN
oJF1027	CGTTCATCAACTAGTTGAAAATGCAG	β-TUBULIN
oJF1028	GAACCTTACAATGCTACTCTGTC	β-TUBULIN
oJF1029	TCTGTACTGCTGAGAACCAC	β-TUBULIN
oJF1113	ATGCCGGACATCTTAAGGAGAATAGCATTTCCTATTGATTTGTCC	*nad9* (downstream)
oJF1144	ATGCCGGACATCTTAAGGAGAAATGGGACCGCGAAGACAAC	*atp9* (downstream)
oJF1145	CCTATGCTTTGCATGAACATCTC	*atp9* (upstream)
oJF1147	GTGTATCCAAGTTTGCGCAAG	TALEN
oJF1298	AGATTACGCTGCTCAGTGCA	HA-tag
oJF1299	GACATTGACTACAAGGATGACGA	FLAG-tag
oJF1300	ATGCCGGACATCTTAAGGAGAGCGACGGTCCCTAACGCT	TALEN
oJF1301	TCCAAAGTACGTTCGTGACG	adapter
oJF1302	TCCAAAGTACGTTCGTGACGCATATCCCAAACTTCTCGCTC	*nad9*

oJF1304	CATACCTATACAAGGGTTCAAG	*nad9*
oJF1305	CCCACAAATGAATAGGAAGCG	Nt mtDNA
oJF1306	GTACAGGGTAGTACTCTTGG	Nt mtDNA
oJF1307	GTTCAATGGTAACGACTTCTTCC	Nt mtDNA
oJF1308	GGTTTGGAACACCAATTCCTC	Nt mtDNA
oJF1309	CGTCTTCCTTTATGCTTCCC	Nt mtDNA
oJF1310	GATGATGCAGGCATCAGAG	Nt mtDNA
oJF1311	TTTCGTAGGCAGCTCTCGA	Nt mtDNA
oJF1312	CTCGATCAACTAGTAGACTTGC	Nt mtDNA
oJF1313	CGTAGCATCATAAGGAGGAC	Nt mtDNA
oJF1314	CCTTATCAGTGAACCGGAAC	Nt mtDNA
oJF1315	ATCAGGTGTCCTCTTGGATG	Nt mtDNA
oJF1316	CACCCACGACAAAATGTCC	Nt mtDNA
oJF1317	CTAGGCATTCAAAAGGAGGGT	Nt mtDNA
oJF1318	CGCTATCTAAAGTAACCTTGCAG	Nt mtDNA
oJF1319	TCCTTGCCTCTGTTGACAC	Nt mtDNA
oJF1320	GAATAGGGCATTTTGGCACTG	Nt mtDNA
oJF1323	AGCGACGGTCCCTAACGCT	TALEN

* with monophosphate group at 5’ end
