# Supplementary material for: Targeted introduction of heritable point mutations into the plant mitochondrial genome
Source: Nat Plants. 2022 Mar 17;8(3):245–56. doi: 10.1038/s41477-022-01108-y (PMC8940627; doi:10.1038/s41477-022-01108-y)
Supplement: Supplementary file 2 — Reporting Summary [file 41477_2022_1108_MOESM2_ESM.pdf]

nature portfolio

Corresponding author(s): Ralph Bock
Last updated by author(s): Feb 7, 2022

Reporting Summary

Nature Portfolio wishes to improve the reproducibility of the work that we publish. This form provides structure for consistency and transparency in reporting. For further information on Nature Portfolio policies, see our Editorial Policies and the Editorial Policy Checklist.

Statistics

For all statistical analyses, confirm that the following items are present in the figure legend, table legend, main text, or Methods section.

n/a	Confirmed	
☐	☒	The exact sample size (n) for each experimental group/condition, given as a discrete number and unit of measurement
☐	☒	A statement on whether measurements were taken from distinct samples or whether the same sample was measured repeatedly
☒	☐	The statistical test(s) used AND whether they are one- or two-sided<br>*Only common tests should be described solely by name; describe more complex techniques in the Methods section.*
☒	☐	A description of all covariates tested
☒	☐	A description of any assumptions or corrections, such as tests of normality and adjustment for multiple comparisons
☐	☒	A full description of the statistical parameters including central tendency (e.g. means) or other basic estimates (e.g. regression coefficient) AND variation (e.g. standard deviation) or associated estimates of uncertainty (e.g. confidence intervals)
☒	☐	For null hypothesis testing, the test statistic (e.g. F, t, r) with confidence intervals, effect sizes, degrees of freedom and P value noted<br>*Give P values as exact values whenever suitable.*
☒	☐	For Bayesian analysis, information on the choice of priors and Markov chain Monte Carlo settings
☒	☐	For hierarchical and complex designs, identification of the appropriate level for tests and full reporting of outcomes
☒	☐	Estimates of effect sizes (e.g. Cohen's d, Pearson's r), indicating how they were calculated

Our web collection on statistics for biologists contains articles on many of the points above.

Software and code

Policy information about availability of computer code

Data collection	NGS datasets were generated on an Illumina's MiSeq system.
Data analysis	Images were analyzed with ImageJ v. 1.52i, DNA sequences with Lasergene v. 14.1.0. The following tools were used for NGS data analysis: FastQC v0.11.9, PEAR v0.9.11, Flexbar v3.5, BWA v0.7.17, SAMtools v1.12, FreeBayes v1.3.2 and SnpEff v4.3k. The Perl code (extract_BaseCovInformation.pl) employed is available under the github repository https://github.com/AxelMacFoly/ngs_perl_scripts/.

For manuscripts utilizing custom algorithms or software that are central to the research but not yet described in published literature, software must be made available to editors and reviewers. We strongly encourage code deposition in a community repository (e.g. GitHub). See the Nature Portfolio guidelines for submitting code & software for further information.

Data

Policy information about availability of data

All manuscripts must include a data availability statement. This statement should provide the following information, where applicable:

- Accession codes, unique identifiers, or web links for publicly available datasets
- A description of any restrictions on data availability
- For clinical datasets or third party data, please ensure that the statement adheres to our policy

The data supporting the findings of this study are available within the paper and its supplementary information files. The NGS datasets were deposited under BioProject ID PRJNA787054 (https://www.ncbi.nlm.nih.gov/bioproject/PRJNA787054). Sequences of plasmids pJF1004, pJF1005 and pJF1006 are accessible via GenBank accession numbers MZ417370, MZ417371 and MZ417372. GenBank accession number NC_006581.1 was used as tobacco mitochondrial reference genome.

Field-specific reporting

Please select the one below that is the best fit for your research. If you are not sure, read the appropriate sections before making your selection.

☒ Life sciences ☐ Behavioural & social sciences ☐ Ecological, evolutionary & environmental sciences

For a reference copy of the document with all sections, see nature.com/documents/nr-reporting-summary-flat.pdf

Life sciences study design

All studies must disclose on these points even when the disclosure is negative.

Sample size	No sample size calculation was performed, as no prior information on the efficiency of the method developed in this work was available. Twenty independent mutant were obtained, confirming that the TALEN-GDM method works with high efficiency.
Data exclusions	No data were excluded from the analysis.
Replication	The mutant isolation procedure developed in the study was successfully performed several times independently, as described in the manuscript. Mutants were obtained in five independent experiments.
Randomization	Randomization was not relevant to the study. Only a single type of biological material was used (i.e., tobacco with proven active mito-TALENs).
Blinding	Blinding was not relevant to the study, because the final read-out (presence/absence of an SNP) is not susceptible to subjective judgment. Experimenters handling the plants have no way of knowing the nucleotide identity at the target locus beforehand, and after sequencing the nucleotide identity is unequivocal as it is a discrete output by an algorithm.

Reporting for specific materials, systems and methods

We require information from authors about some types of materials, experimental systems and methods used in many studies. Here, indicate whether each material, system or method listed is relevant to your study. If you are not sure if a list item applies to your research, read the appropriate section before selecting a response.

Materials & experimental systems

n/a	Involved in the study	
☐	☒	Antibodies
☒	☐	Eukaryotic cell lines
☒	☐	Palaeontology and archaeology
☒	☐	Animals and other organisms
☒	☐	Human research participants
☒	☐	Clinical data
☒	☐	Dual use research of concern

Methods

n/a	Involved in the study	
☒	☐	ChIP-seq
☒	☐	Flow cytometry
☒	☐	MRI-based neuroimaging

Antibodies

Antibodies used	Primary antibodies: anti-Nad9 (used at 1:20,000 dilution), anti-Cox1 (used at 1:10,000 dilution), and anti-CA2 (used at 1:10,000 dilution). For these polyclonal primary antibodies, there are no supplier name, catalog, clone or lot number, but see references listed below for more information. Secondary antibody: anti-rabbit-HRP conjugate from Sigma (A0545) used at 1:10,000 dilution.
Validation	Details on the antibodies and their specificity are found in references 66 (anti-Nad9), 67 (anti-Cox1) and 68 (anti-CA2).
