# Supplementary figures and images for: Targeted introduction of heritable point mutations into the plant mitochondrial genome
Source: Nat Plants. 2022 Mar 17;8(3):245–56. doi: 10.1038/s41477-022-01108-y (PMC8940627; doi:10.1038/s41477-022-01108-y)

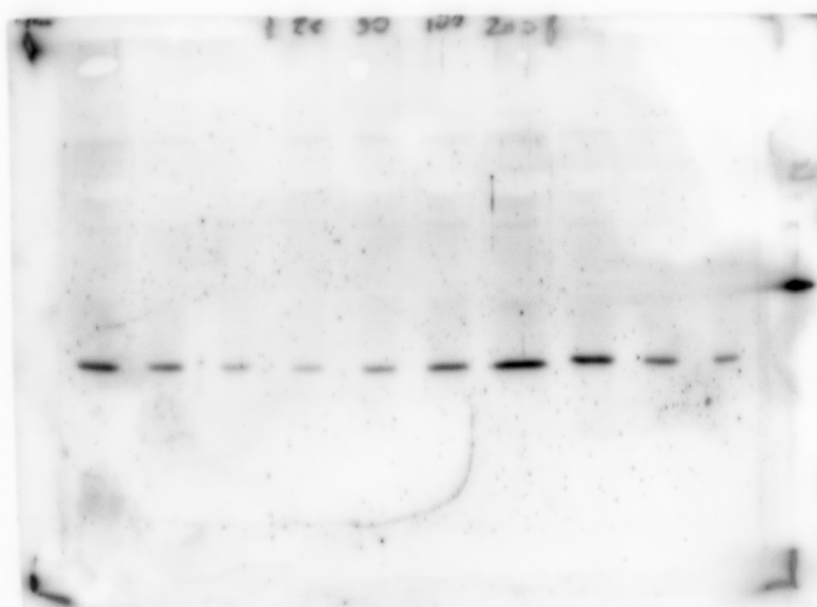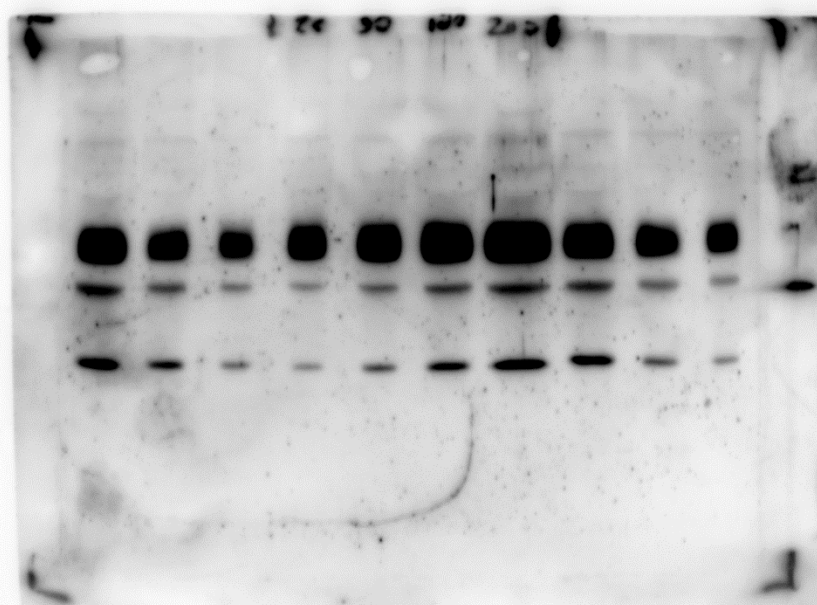

Supplement: Source Data Extended Data Fig. 9 — Unprocessed western blots. [file 41477_2022_1108_MOESM4_ESM.pdf]
